# H3K27me3-H3K4me1 transition at bivalent promoters instructs lineage specification in development

**DOI:** 10.1186/s13578-023-01017-3

**Published:** 2023-03-29

**Authors:** Yue Yu, Xinjie Li, Rui Jiao, Yang Lu, Xuan Jiang, Xin Li

**Affiliations:** 1 School of Medicine, Shenzhen Campus of Sun Yat-sen University, Shenzhen, China; 2 The Seventh Affiliated Hospital of Sun Yat-sen University, Shenzhen, China; 3 Guangdong Provincial Key Laboratory of Digestive Cancer Research, The Seventh Affiliated Hospital of Sun Yat-sen University, Shenzhen, China

**Keywords:** H3K27me3-H3K4me1 transition, Bivalent promoter, Tissue-specific genes, LSD1, Neural ectoderm differentiation

## Abstract

**Background:**

Bivalent genes, of which promoters are marked by both H3K4me3 (trimethylation of histone H3 on lysine 4) and H3K27me3 (trimethylation of histone H3 on lysine 27), play critical roles in development and tumorigenesis. Monomethylation on lysine 4 of histone H3 (H3K4me1) is commonly associated with enhancers, but H3K4me1 is also present at promoter regions as an active bimodal or a repressed unimodal pattern. Whether the co-occurrence of H3K4me1 and bivalent marks at promoters plays regulatory role in development is largely unknown.

**Results:**

We report that in the process of lineage differentiation, bivalent promoters undergo H3K27me3-H3K4me1 transition, the loss of H3K27me3 accompanies by bimodal pattern loss or unimodal pattern enrichment of H3K4me1. More importantly, this transition regulates tissue-specific gene expression to orchestrate the development. Furthermore, knockout of *Eed* (Embryonic Ectoderm Development) or *Suz12* (Suppressor of Zeste 12) in mESCs (mouse embryonic stem cells), the core components of Polycomb repressive complex 2 (PRC2) which catalyzes H3K27 trimethylation, generates an artificial H3K27me3-H3K4me1 transition at partial bivalent promoters, which leads to up-regulation of meso-endoderm related genes and down-regulation of ectoderm related genes, thus could explain the observed neural ectoderm differentiation failure upon retinoic acid (RA) induction. Finally, we find that lysine-specific demethylase 1 (LSD1) interacts with PRC2 and contributes to the H3K27me3-H3K4me1 transition in mESCs.

**Conclusions:**

These findings suggest that H3K27me3-H3K4me1 transition plays a key role in lineage differentiation by regulating the expression of tissue specific genes, and H3K4me1 pattern in bivalent promoters could be modulated by LSD1 via interacting with PRC2.

**Supplementary Information:**

The online version contains supplementary material available at 10.1186/s13578-023-01017-3.

## Background

In 2006, Bernstein et al. firstly identified a subset of genes, known as bivalent genes, of which promoter regions are marked by both a repressive histone mark trimethylation of histone H3 on lysine 27 (H3K27me3) and an active histone mark trimethylation of histone H3 on lysine 4 (H3K4me3) in mouse embryonic stem cells (mESCs) [1]. Subsequently, bivalent genes were also found in some other types of stem cells including human embryonic stem cells (hESCs), induced pluripotent stem cells (iPSCs), hematopoietic stem cells, and embryonic tissues including human fetal brain, heart, liver [2–4]. The co-occurrence of these two opposing modifications maintains the promoter in a poised state in ES cells, allowing a transcriptional activation upon differentiation signal, or remaining repression during development [5–9].

Polycomb repressive complex 2 (PRC2) is responsible for catalyzing the trimethylation of H3K27, which comprises four core members, including catalytic subunit Enhancer of Zeste homologue 2 (EZH2) or its paralogue EZH1, the cofactor of catalytic subunit Embryonic Ectoderm Development (EED), the stabilizing factor Suppressor of Zeste 12 (SUZ12) and the nucleosomes binding factor retinoblastoma-binding protein 4/7 (RBBP4 or RBBP7) [10]. H3K4 is mainly methylated by Complex Proteins Associated with Set1 (COMPASS), and there are six methyltransferases that methylates H3K4 in mammals, including SET1A (also known as SETD1A, KMT2F), SET1B (also known as SETD1B, MKT2G), mixed lineage leukemia 1 (MLL1; also known as KMT2A), MLL2 (also known as KMT2B), MLL3 (also known as KMT2C) and MLL4 (also known as KMT2D) [11–13]. Each of these methyltransferases in combination with different subunit composition form a distinct COMPASS complex with different function [11–13]. The MLL2 is identified as the major lysine methyltransferases (KMTs) for H3K4me3 at bivalent promoters [12, 13].

The bivalent genes are thought to play key roles in embryonic development, cell differentiation and tumorigenesis. Studies in stem cells and mouse animal models have shown that core members of the ﻿COMPASS and PRC2 complexes, which are closely linked to bivalent promoter formation, have significant influences on embryonic development [10, 12, 13]. For example, conventional knockout of *Eed*, *Ezh2, Suz12* (genes coding for members of the PRC2 complex), *Set1a, Set1b, Mll1 or Mll2* (genes coding for the COMPASS complex) in mice results in early embryonic lethality [13, 14]. Mutation or aberrantly expression of core members of the COMPASS or PRC2 complex were also frequently observed in various types of tumors [12, 15, 16]. Consistently, cancer cells are characterized by DNA methylation abnormalities, mainly global hypomethylation and locus-specific hypermethylation [17]. Remarkably, multiple studies showed that DNA hypermethylation in tumors can take place precisely at the bivalent promoters [18–21]. Several of these bivalent genes are reported to act as tumor-suppressor genes in cancer [22].

H3K4me3 usually features the transcriptional active promoters, while H3K4me1 is enriched at primed enhancers to fine-tune its activity. The MLL3/MLL4 complexes are able to mono-methylate H3K4, together with H3K27 acetylation, to form an active enhancer landscape [12, 13, 23]. Conversely, LSD1-mediated H3K4me1 demethylation silences the enhancer of pluripotency genes to allow the differentiation of various stem cells, such as mouse ESCs [24], cancer stem cells [25], endocrine progenitor cells [26] and others. Other than enhancers, H3K4me1 also is present at promoters [27] where it exhibits two different distribution patterns: a bimodal pattern at active promoters flanked by H3K4me3, or a unimodal pattern that coincides with H3K4me3 and H3K27me3 at poised promoters [28, 29]. In addition, H3K4me1 is found to predispose DNA methylation encroachment at CpG island shores in cancers, leading to repression of genes in vicinity [30]. Taken together, previous studies on the role of H3K4me1 at promoters were limited to the correlation of its distribution pattern and gene expression, while the regulation on H3K4me1 at bivalent promoters and its role during development are largely unknown.

In this study, we found that H3K4me1 in combination with H3K4me3 can represent most traditional bivalent promoters, implying most traditional bivalent promoters are actually trivalent promoters marked by both H3K4me1 and bivalent marks. Furthermore, during tissue and cell development, the bivalent promoters undergo a H3K27me3-H3K4me1 transition: the loss of H3K27me3 accompanies by bimodal pattern loss or unimodal pattern enrichment of H3K4me1, and this transition regulates tissue-specific genes expression. We proposed a model where PRC2 interacts with LSD1 to specify early neural ectoderm development in mESCs by inducing the H3K27me3-H3K4me1 transition at the promoter of bivalent genes that regulate meso-endoderm and neural ectoderm fate. Finally, our results assign a new role to H3K4me1, a conditional repressor at bivalent promoters during development, and further uncover a layer of epigenetic regulation mediated by PRC2 and LSD1 through the H3K27me3-H3K4me1 transition at bivalent promoters during lineage specification at early stages of development.

## Results

### H3K4me1 in combination with H3K4me3 is able to partition promoters and predict bivalent promoters

Considering that it has been previously reported that both H3K4me1 and H3K27me3 are present at the promoter [1, 27–29], and are associated with gene repression [1, 29], we sought to explore whether there are distinct roles for H3K4me1 and H3K27me3 at promoters. We analyzed chromatin immunoprecipitation sequencing (ChIP-seq) data of H3K4me1, H3K4me3, H3K27me3 in hESCs from public database ENCODE. The two repressive marks H3K4me1 or H3K27me3, in combination with the active mark H3K4me3 respectively, was used to classify the promoter CpG islands (CGIs). Through k-means clustering, promoter CGIs were categorized into three clusters based on the distribution patterns of traditional bivalent marks (H3K4me3 and H3K27me3) or non-traditional bivalent marks (H3K4me1 and H3K4me3), respectively (Additional file 1: Fig. S1A). We found considerable overlaps between the clusters defined based on the two different bivalent mark combinations described above, respectively (Additional file 1: Fig. S1A). For example, 94% of the 2,789 non-traditional bivalent promoter CGIs were also present in the traditional bivalent promoter CGIs clusters, and comprise 73% of the traditional bivalent promoter CGIs clusters. Together, these results suggest that H3K4me1 in combination with H3K4me3 is able to distinguish bivalent and non-bivalent promoter CGIs in hESCs, implying that H3K4me1 overlaps with H3K27me3 at bivalent promoter CGIs. Consistently, H3K4me1 occupies H3K27me3 marked promoters in a wide variety of cells, such as hESCs, muscle cells and germs cells [28, 31, 32]. In other words, most traditional bivalent promoters are trivalent promoters, co-labelled by all three marks H3K4me1, H3K4me3 and H3K27me3.

**Fig. 1:**
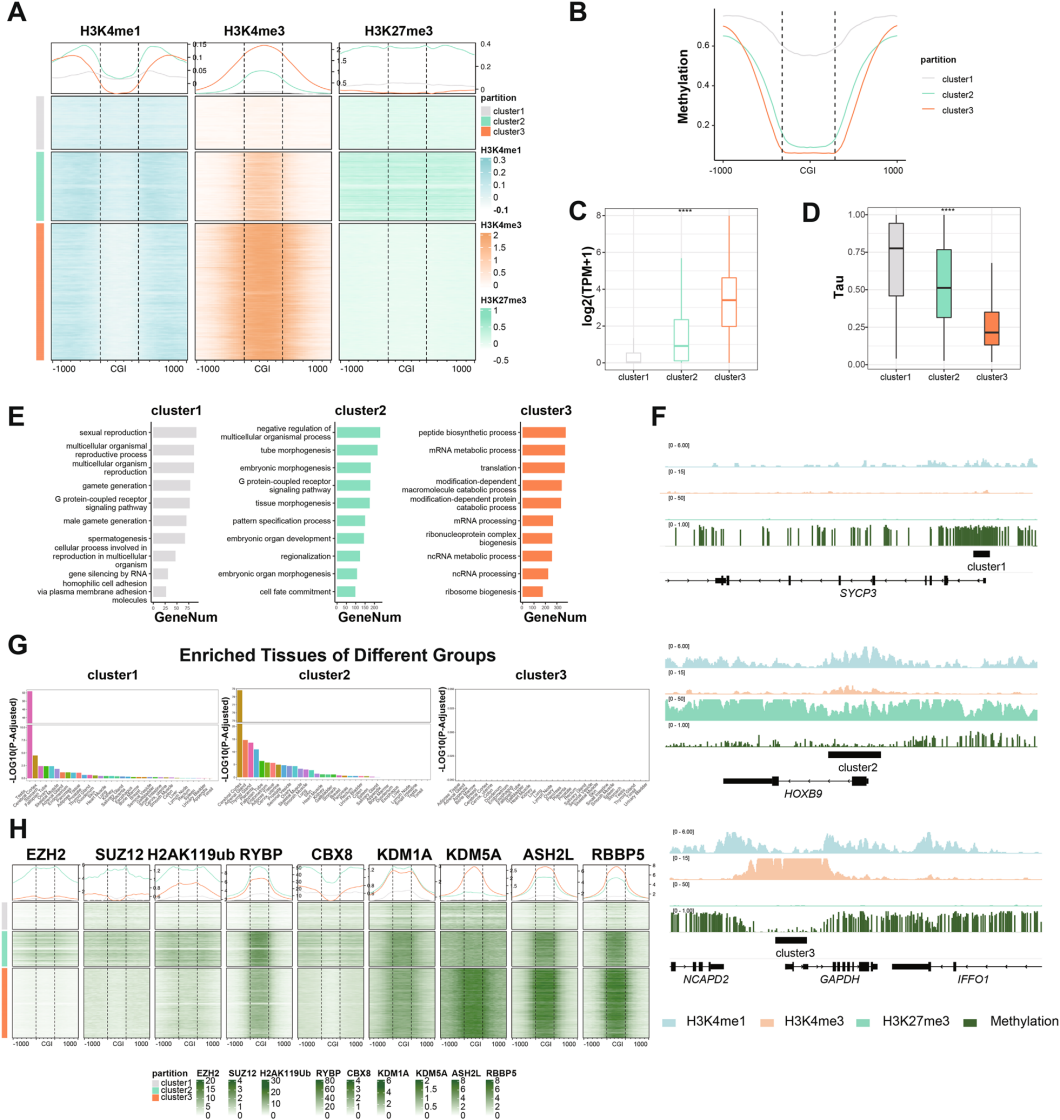
H3K4me1 in combination with H3K4me3 is able to partition promoters and predict bivalent promoters in hESCs. **A** Heatmaps and average line plots showing histone modification patterns of promoter CGIs and their shores in hESCs. Each line represents a single CpG island. **B** Average methylation patterns across CGIs and their shores of promoters in three clusters in hESCs as in (**A**).** C** Boxplot showing gene expression level in three clusters as in (**A**). Significance was examined with Kruskal–Wallis rank-sum test, ****p value < 0.0001.** D** Boxplot showing tissue-specific score (Tau) of genes in three clusters. Significance was examined with Kruskal–Wallis rank-sum test, ****p value < 0.0001.** E** Histograms showing GO-term enrichment for genes involved in three clusters as in (**A**).** F** Integrative Genomics Viewer (IGV) browser track showing histone modifications and DNA methylation patterns at promoter CGIs of three different clusters as in (**A**), ﻿respectively.** G** Tissue enrichment analysis for genes involved in three clusters as in (**A**).** H** Heatmaps and average line plots showing H3K4 and H3K27 methylation related HMTs (EZH2, SZU12 for H3K27; ASH2L, RBBP5 for H3K4; RYBP, CBX8 for H2AK119ub) and HDMTs (KDM1A, KDM5A for H3K4) patterns of promoter CGIs and their shores of three clusters respectively. Each line represents a single CpG island

Considering the significant role of H3K4me1 at promoters, promoter CGIs were repartitioned into three clusters based on the distribution of all three histone methylation marks. We subsequently combined public data of DNA methylation, gene expression level and tissue/cell type-specific expression pattern to fully characterize the three different clusters of promoters in more detail. Cluster 1 promoters show low enrichment of both H3K4 and H3K27 modifications (Fig. 1A) and high levels of DNA methylation (Fig. 1B), and are associated with repressive genes (Fig. 1C). Cluster 2 promoters are traditional bivalent promoters with elevated H3K4me3 and H3K27me3 level, while H3K4me1 shows an untypical bimodal pattern with high H3K4me1 level at CGIs (Fig. 1A). Consistent with previous research, promoters of this cluster are generally hypomethylated (Fig. 1B). Expression of these genes are highly tissue-specific while repressed in majority of other cells (Fig. 1C, D). Cluster 3 promoters show significantly enriched H3K4me3, while H3K4me1 exhibits a typical bimodal pattern with low H3K4me1 at CGIs (Fig. 1A). Promoters of this cluster show low levels of DNA methylation (Fig. 1B). Genes of this cluster show high levels of expression (Fig. 1C). Similar histone modifications patterns were also observed in mESCs, indicating that the distribution patterns of H3K4me1, H3K4me3 and H3K27me3 at promoters are conserved between human and mouse (Additional file 1: Fig. S1B).

To further investigate the biological significance of the genes among three different clusters, Gene Ontology (GO) enrichment analysis was performed. Genes involved in cluster1 show enrichment in classical reproduction-related pathways such as gamete generation and ﻿spermatogenesis (Fig. 1E; Additional file 1: Fig. S1C)*.* For example, Synaptonemal complex protein 3 (SYCP3) gene encodes a component of the synaptonemal complex, which formed between homologous chromosomes in the prophase of meiosis [33]. It shows low enrichment of all three marks and DNA hypermethylation at promoter locus in hESCs (Fig. 1F). Genes involved in cluster 3 are mainly house-keeping genes regulating RNA and protein metabolism (Fig. 1E; Additional file 1: Fig. S1C). For example, *GAPDH,* one of the enzymes involved in carbohydrate metabolism [34] is highly-expressed in hESCs. The promoter of *GAPDH* gene shows high enrichment of H3K4me3 and low levels of DNA methylation (Fig. 1F). In contrast to these non-bivalent clusters (cluster 1 and cluster 3), genes involved in bivalent cluster 2 regulate cell fate commitment, lineage differentiation and development (Fig. 1E; Additional file 1: Fig. S1C). For example, *HOX* genes, encode evolutionarily conserved transcription factors that show strict temporal and spatial specific pattern to establish morphogenesis of the vertebrate embryos. Promoters of *HOX* genes are marked by bivalent modifications in embryonic stem cells (ESCs) [35] (Fig. 1F). Consistently, tissue enrichment analysis showed that genes in cluster 1 are predominantly enriched in testis tissue, genes in cluster 3 do not show any tissue-specific enrichment, while genes in cluster 2 showed an intermediate tissue enrichment pattern, enriched at a medial level in multiple tissues (Fig. 1G).

Meanwhile, to further support these results, we analyzed ChIP-seq data of major histone-modifying enzymes including HMTs (histone methyltransferases), such as EZH2、SUZ12 and RBBP5, and HDMTs (histone demethylases), such as KDM1A. The results showed that distribution patterns of histone-modifying enzymes across the whole genome are consistent with corresponding histone modifications (Fig. 1H; Additional file 1: Fig. S1D). For example, EZH2 and SUZ12, the core components of PRC2 complexes that generate H3K27me3, are mainly distributed on the promoters in cluster 2, which are highly marked by H3K27me3. RBBP5 (retinoblastoma binding protein 5) and ASH2L (absent, small, or homeotic 2-like), core subunits of COMPASS families that methylate H3K4, are distributed mainly on promoters in cluster 2 and 3, which show enriched H3K4 methylation.

Together, these findings suggest that H3K4me1 considerably represent H3K27me3 in partitioning promoter CGIs in ESCs. H3K4me1 in combination with H3K4me3 predict most of traditional bivalent promoters. Moreover, the distribution pattern of H3K4me1 alone is able to predict bivalent promoters: an untypical bimodal pattern with high H3K4me1 at CGIs was observed across bivalent promoters, while active promoters display a typical bimodal pattern with low H3K4me1 at CGIs (Fig. 1A). Consistently, a previous study showed that unimodal H3K4me1 pattern correlates strongly with poised promoters marked by both H3K4me3 and H3K27me3 in germs cells [29].

### Bivalent promoter CGIs undergo H3K27me3-H3K4me1 transition during development

**Fig. 2:**
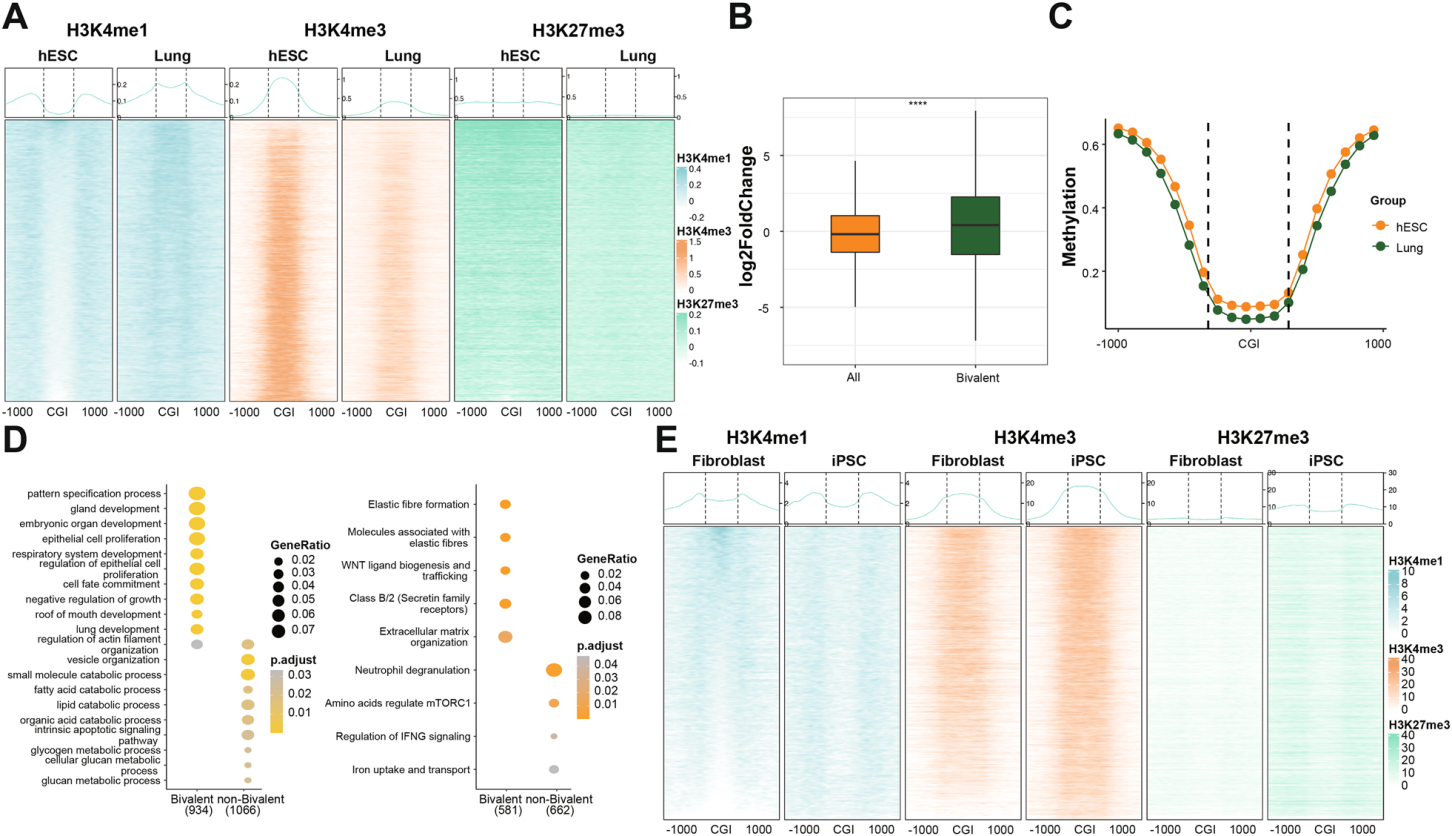
Bivalent promoter CGIs undergo H3K27me3-H3K4me1 transition during development. **A** Heatmaps and average line plots showing H3K4me1, H3K4me3 and H3K27me3 patterns of promoter CGIs and their shores in bivalent cluster in hESC and lung. Each line represents a single CpG island. **B** Boxplot showing expression differences (log2FoldChange) between lung and hESC of genes in all genes (ALL) and bivalent cluster genes (Bivalent), ﻿respectively. Significance was examined with Wilcoxon rank-sum test, ****p value < 0.0001. **C** Average methylation patterns across CGIs and their shores of bivalent cluster in hESCs and lung. **D** Function enrichment analysis of all up-regulated genes between bivalent (Bivalent) and non-bivalent (non-Bivalent) genes in lung. Left panel: GO enrichment; right panel: Reactome pathway enrichment.** E** Heatmaps and average line plots showing H3K4me1, H3K4me3 and H3K27me3 patterns of promoter CGIs and their shores in bivalent cluster between human BJ foreskin fibroblast (hBJ)s (Fibroblast) and the iPSC cells reprogrammed from hBJ. Each line represents a single CpG island

Considering bivalent genes are predicted by H3K4me1 pattern and play significant roles in embryonic development, we hypothesized that the alteration of H3K4me1 pattern correlates with gene expression changes and plays a significant role in development. To this end, the dynamics of H3K4me1 pattern during embryonic development was studied. In the developmental process of multiple human tissues such as lung, liver, stomach, small intestine, spleen, pancreas, and fibroblasts, loss of H3K27me3 at bivalent promoter CGIs was accompanied by bimodal pattern loss or unimodal pattern enrichment of H3K4me1 (Fig. 2A; Additional file 2: Fig. S2A). Although the transition was observed in all these six tissues, the extent of the H3K27me3-H3K4me1 transition among different tissues was heterogeneous. The extent of the H3K27me3-H3K4me1 transition in liver, pancreas, stomach, spleen and small intestine was less than that in lung (Fig. 2A; Additional file 2: Fig. S2A). In addition, a decrease of H3K4me3 modifications was also observed at bivalent promoter CGIs during development, consistent with previous report that H3K4me1 and H3K4me3 are usually mutually exclusive [28] (Fig. 2A; Additional file 2: Fig. S2A). Furthermore, this H3K27me3-H3K4me1 transition was not observed at enhancers (Additional file 2: Fig. S2B). As excepted, the expression of genes in bivalent cluster 2 was significantly up-regulated during development (*p* value < 0.0001, Wilcoxon rank-sum test; Fig. 2B), while the DNA methylation at promoters was not altered (Fig. 2C). Functional enrichment analysis of these up-regulated genes during development shows that these genes are mainly involved in ﻿development-related pathways such as cell fate commitment and ﻿embryonic organ specification (Fig. 2D). Taken altogether, these results suggest that the H3K27me3-H3K4me1 transition regulates expression of genes that orchestrate tissues development. Consistently, this H3K27me3-H3K4me1 transition was also observed during mouse tissues development (Additional file 2: Fig. S2C), indicating this H3K27me3-H3K4me1 transition during development is conserved in mammals.

Next, to explore whether this H3K27me3-H3K4me1transition during development is reversible, we analyzed the distribution of H3K4me1, H3K4me3 and H3K27me3 modifications at cluster 2 promoters of human BJ foreskin fibroblasts (hBJs), mouse embryonic fibroblasts (MEFs) and the included pluripotent stem cells (iPSCs) reprogrammed from hBJs and MEFs [36, 37]. Notably, we found that the H3K27me3-H3K4me1 transition is reversed when hBJs or MEFs were reprogrammed into iPSCs (Fig. 2E and Additional file 2: Fig. S2D). These results suggest that the H3K27me3-H3K4me1 transition at bivalent promoter CGIs closely correlate with cells on differentiation trajectory.

Taken together, the H3K27me3-H3K4me1 transition is conserved in mammals, and closely linked with the specification of the cell fate and lineages priming at early stages of development. Furthermore, the H3K27me3-H3K4me1 transition is reversible upon cell fate reprogramming.

### H3K27me3-H3K4me1 transition influences tissue-specific gene expression

**Fig. 3:**
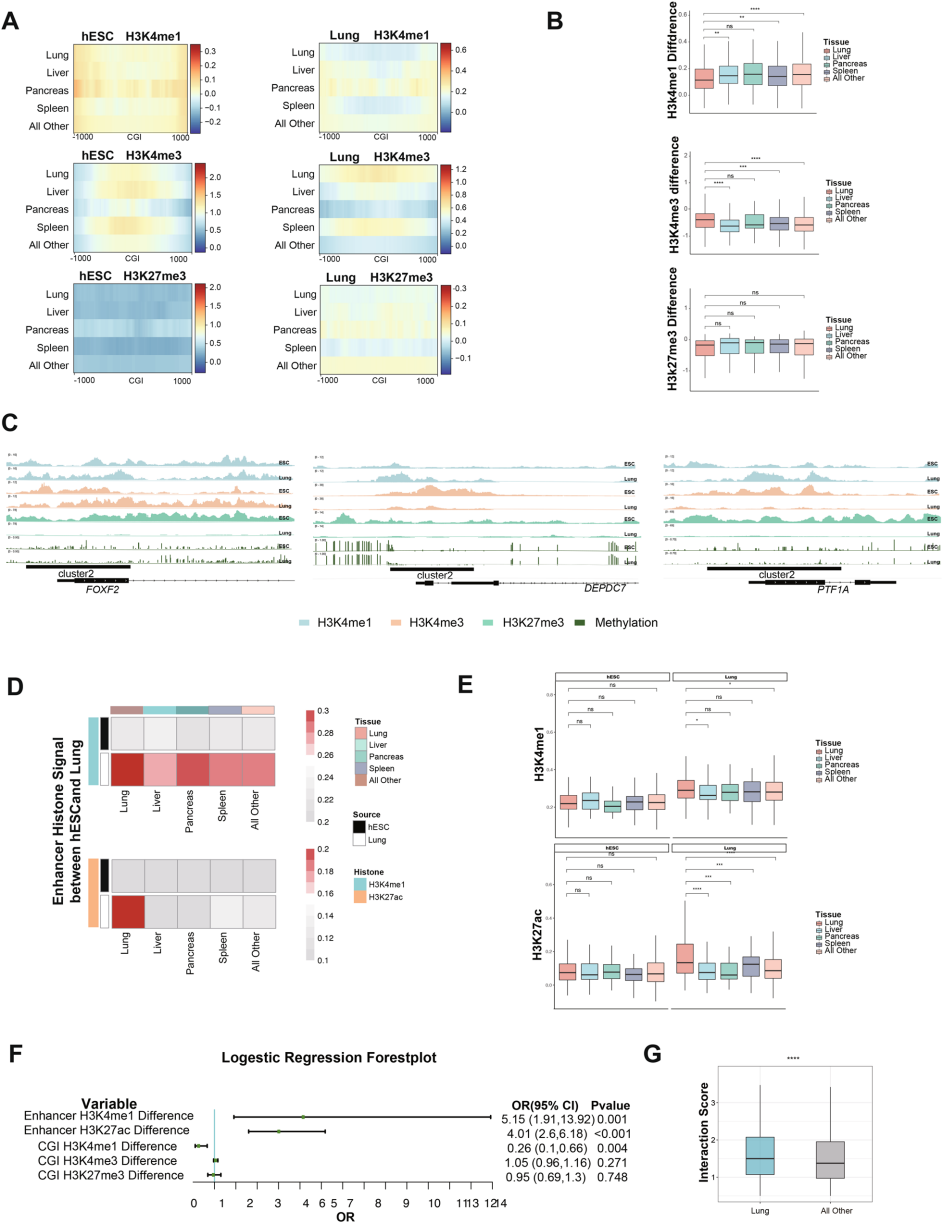
H3K27me3-H3K4me1 transition influences tissue-specific gene expression. **A** Heatmaps showing patterns of H3K4me1, H3K4me3 and H3K27me3 of each tissue-specific bivalent genes at promoter CGIs and their shores in hESCs and lung. Lung: tissue-specific bivalent genes of lung; Liver: tissue-specific bivalent genes of liver; Pancreas: tissue-specific bivalent genes of pancreas; Spleen: tissue-specific bivalent genes of spleen; All Other: all the other tissues without lung. Each line represents a single CpG island. **B** Boxplots showing differences of H3K4me1, H3K4me3 and H3K27me3 between hESC and lung of each tissue-specific bivalent genes at promoter CGIs. Lung: tissue-specific bivalent genes of lung; Liver: tissue-specific bivalent genes of liver; Pancreas: tissue-specific bivalent genes of pancreas; Spleen: tissue-specific bivalent genes of spleen; All Other: all the other tissues without lung. Significance was examined with *t*-test, *p value < 0.05, **p value < 0.01, ****p value < 0.0001﻿, ns: not significantly. **C** IGV browser track showing the H3K27me3-H3K4me1 transition from hESCs to lung at bivalent promoter CGIs. Lung tissue-specific bivalent gene (*FOXF2*) undergoes lower unimodal enrichment of H3K4me1 than non-lung tissue-specific bivalent genes (*PTF1A* and *DEPDC7*). **D** Heatmaps showing patterns of H3K4me1 and H3K27ac at enhancers of each tissue-specific bivalent genes in hESCs and lung, respectively. Lung: tissue-specific bivalent genes of lung; Liver: tissue-specific bivalent genes of liver; Pancreas: tissue-specific bivalent genes of pancreas; Spleen: tissue-specific bivalent genes of spleen; All Other: all the other tissues without lung. Each line represents a single enhancer. **E** Boxplots showing levels of H3K4me1 and H3K27ac at enhancers of each tissue-specific bivalent genes in hESCs and lung, respectively. Lung: tissue-specific bivalent genes of lung; Liver: tissue-specific bivalent genes of liver; Pancreas: tissue-specific bivalent genes of pancreas; Spleen: tissue-specific bivalent genes of spleen; All Other: all the other tissues without lung. Significance was examined with *t*-test, *p value < 0.05, ****p value < 0.0001, *ns* not significantly. **F** Forest plot showing association between the differences of the level of enhancer H3K4me1, enhancer H3K27ac, CGI H3K4me1, CGI H3K4me3 and CGI H3K27me3 between hESC and lung and tissue specificity. **G** Boxplot showing interaction score between promoter and enhancer of lung tissue-specific and all other tissue (lung not included)-specific bivalent genes in lung. Significance was examined with Wilcoxon rank-sum test, ****p value < 0.0001

Next, we sought to investigate how the H3K27me3-H3K4me1 transition affects the tissue development through regulating expression of bivalent genes. Tissue-specific bivalent genes were filtered out from the Genotype-Tissue Expression (GTEx) database in a way reported by Teng et al. [38] and subjected for the following analysis across multiple tissues. In each tissue, the pattern and level of three histone modifications at promoter CGIs of self-tissue-specific bivalent genes and other tissue-specific bivalent genes were compared. We found no significant differences of H3K4me1, H3K4me3 and H3K27me3 modifications at promoter CGIs between self-tissue-specific bivalent genes and other tissue-specific bivalent genes in hESCs, both groups of genes undergo the H3K27me3-H3K4me1 transition during development (Fig. 3A). However, in each specific tissue, self-tissue-specific bivalent genes exhibit lower enrichment of unimodal H3K4me1 at promoter CGIs than other tissue-specific bivalent genes, while the level of H3K27me3 modification is comparable at promoter CGIs (*p* < 0.05, *t*-test; Fig. 3B and Additional file 3: Fig. S3A). For example, the lung tissue-specific gene *FOXF2,* that can transcriptionally activate several other lung-specific genes to ensure lung function [39], shows lower unimodal enrichment of H3K4me1 at promoter CGIs compared with bivalent genes functioned in other tissues, such as *PTF1A* and *DEPDC7,* which specifically instructs pancreas and liver development, respectively [40–42] (Fig. 3C). Collectively, the differential level of unimodal H3K4me1 between self-tissue-specific bivalent genes and other tissue-specific bivalent genes after H3K27me3-H3K4me1 transition determines the expression level of these genes. After the loss of H3K27me3, the lower level of H3K4me1 unimodal reserves higher transcription level of self-tissue-specific bivalent genes compared with other tissue-specific bivalent genes during development.

Other than methylation, acetylation of histones also regulates the gene transcription. Acetylation on lysine 27 of histone 3 (H3K27ac) on enhancer region is associated with transcriptional activation [23]. The histone modification landscapes are analyzed comprehensively to dissect the regulation mechanisms of tissue-specific bivalent genes. Higher occupancy by the active enhancer marks H3K4me1 and H3K27ac was observed at tissue-specific bivalent gene enhancers in each specific tissue such as lung, liver, and pancreas compared with that in hESCs (Fig. 3D, E; Additional file 3: Fig. S3B). Next, logistic regression analysis was performed to examine the contribution of enhancer H3K4me1, enhancer H3K27ac, promoter H3K4me1, promoter H3K4me3 and promoter H3K27me3 to tissue specificity of bivalent genes. Results showed that the level of enhancer H3K27ac, enhancer H3K4me1 and promoter H3K4me1 during development synergistically contribute the tissue specificity of bivalent genes in each specific tissue (Fig. 3F). It is well established that spatial interactions between enhancers and promoters affect gene expression [43], we next asked whether promoter-enhancer interaction also contributes to the regulation of tissue-specific bivalent gene expression during development. The analysis of interaction between enhancers and promoters based on the Enhancer Atlas database showed that promoter-enhancer interactions at tissue-specific bivalent genes are significantly stronger than those at other bivalent genes in each specific tissue (*p* value < 0.0001, Wilcoxon rank-sum test; Fig. 3G; Additional file 3: Fig. S3C).

Collectively, our results showed that dynamic landscapes of three histone modification events, including H3K27me3-H3K4me1 transition, existence of enhancer H3K27ac and enhancer H3K4me1, as well as promoter-enhancer interaction, work together to regulate the expression of tissue-specific bivalent genes in multiple tissues and lineage priming.

### An artificial H3K27me3-H3K4me1 transition regulates the ESCs differentiation

To confirm that H3K27me3-H3K4me1 transition determines the expression of bivalent genes in the process of tissue specification, we generated an artificial H3K27me3-H3K4me1transition through inactivating *Eed* and *Suz12* in mESCs.

**Fig. 4:**
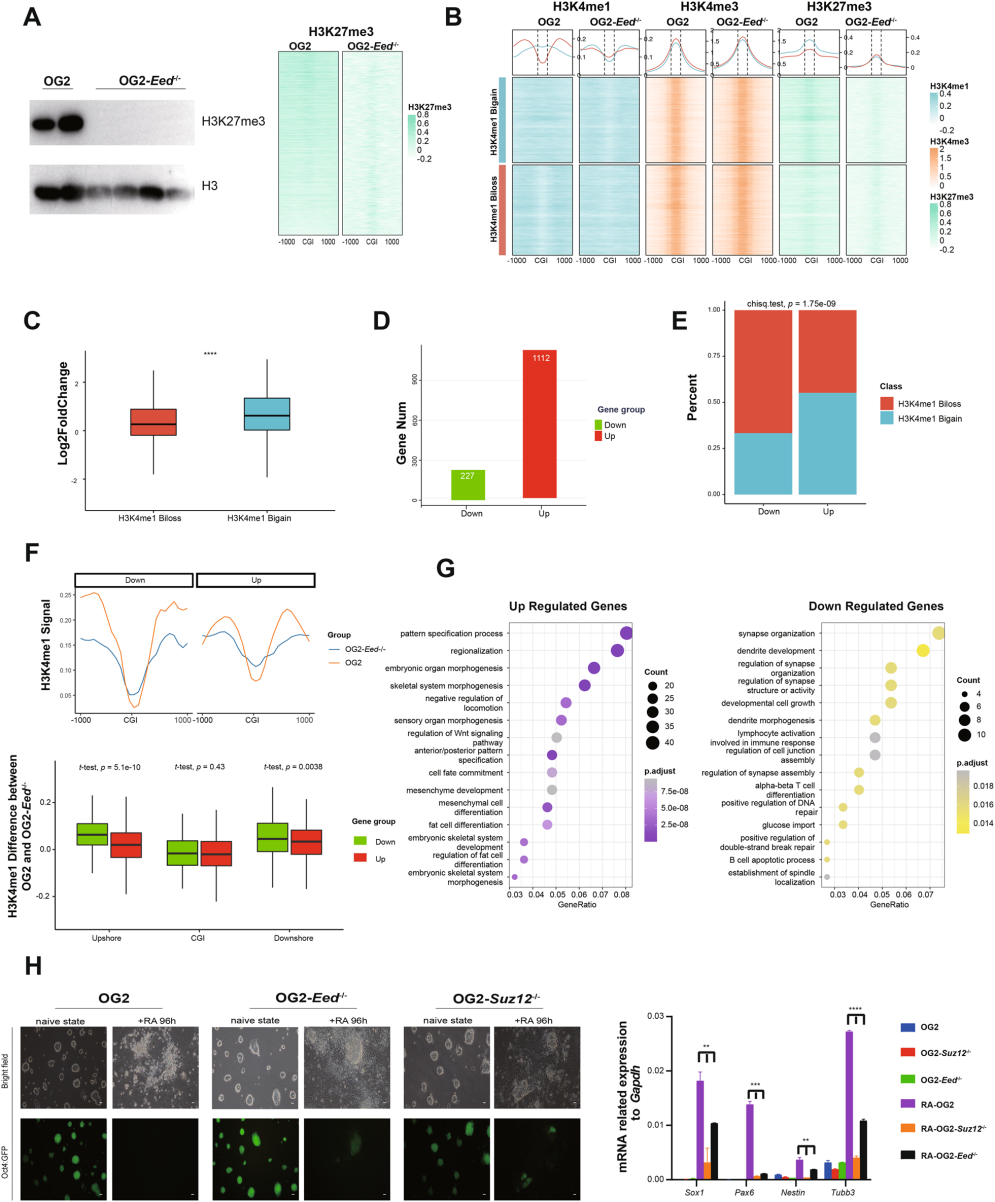
An artificial H3K27me3-H3K4me1 transition regulates the ESCs differentiation. **A** Left panel: western blotting analysis of H3K27me3 modification in WT and *Eed*^*−/−*^ OG2 mESCs. H3 as the loading control; Right panel: heatmap showing H3K27me3 patterns of promoter CGIs and their shores in WT and *Eed*^*−/−*^ OG2 mESCs. OG2: WT OG2 mESCs; OG2 *Eed*^*−/−*^: *Eed*^*−/−*^ OG2 mESCs. Each line represents a single CpG island. **B** Heatmaps and average line plots showing H3K4me1, H3K4me3 and H3K27me3 patterns at promoter CGIs and their shores of two different groups (H3K4me1 bimodal-loss group and bimodal-gain group) in WT and *Eed*^*−/−*^ OG2 mESCs. Each line represents a single CpG island. H3K4me1 Biloss: bimodal-loss group; H3K4me1 Bigain: bimodal-gain group. **C** Boxplot showing expression alteration (log2FoldChange) of bivalent genes in H3K4me1 biloss group and H3K4me1 bigain group in *Eed*^*−/−*^ OG2 mESCs compared with WT OG2 mESCs. H3K4me1 Biloss: bimodal-loss group; H3K4me1 Bigain: bimodal-gain group. Significance was examined with Wilcoxon rank-sum test, ****p value < 0.0001). **D** Histogram showing the number of up-regulated (Up) and down-regulated (Down) bivalent genes in *Eed*^*−/−*^ OG2 mESCs. **E** Histogram showing the percentage of H3K4me1 biloss group genes and H3K4me1 bigain group genes in the up-regulated (Up) or down-regulated (Down) genes in *Eed*^*−/−*^ OG2 mESCs compared with WT OG2 mESCs, respectively. H3K4me1 Biloss: bimodal-loss group; H3K4me1 Bigain: bimodal-gain group. Significance level was determined using *χ2* tests. *p* value  = 1.75e−09. **F** Average line plots showing H3K4me1 pattern of promoter CGIs and their shores in WT and *Eed*^*−/−*^ OG2 mESCs between up-regulated (Up) and down-regulated (Down) bivalent genes in H3K4me1 bimodal-loss group. Boxplot showing the differences of normalized H3K4me1 coverage at promoter CGIs and their shores between WT and *Eed*^*−/−*^ OG2 mESCs of up-regulated (Up) and down-regulated (Down) bivalent genes in H3K4me1 bimodal-loss group. OG2: WT OG2 mESCs; OG2 *Eed*^*−/−*^: *Eed*^*−/−*^ OG2 mESCs. Significance was examined with *t*-test, *p* value = ﻿5.1e-10, 0.43, 0.0038. **G** GO enrichment analysis of up-regulated and down-regulated bivalent genes in H3K4me1 bimodal-loss group. **H** Images showing the morphology of mESC cells after RA treatment. Right panel: qRT-PCR analysis of lineage markers. WT mESCs is OG2 mESCs with GFP expression controlled by *Oct4* promoter (Oct4: GFP). Scale bar, 100 μm. Significance was examined with *t*-test, **p value < 0.01, ***p value < 0.001, ****p value < 0.0001. The data represent mean ± SD from three repeats

Subsequent ChIP-seq and western blot analysis confirmed that H3K27me3 modification on chromatin was completely abolished in *Eed*^*−/−*^ and *Suz12*^*−/−*^ OG2 mESCs (Fig. 4A; Additional file 4: Fig. S4A). H3K4me3 pattern was generally reserved in both *Eed*^*−/−*^ and *Suz12*^*−/−*^ OG2 mESCs (Fig. 4B; Additional file 4: Fig. S4B). As expected, the abolishment of H3K27me3 initiates an artificial H3K27me3-H3K4me1 transition in both *Eed*^*−/−*^ and *Suz12*^*−/−*^ OG2 mESCs (Fig. 4B; Additional file 4: Fig. S4B). All the bivalent genes are subsequently divided into two groups, named as bimodal-loss group and bimodal-gain group, respectively (Fig. 4B; Additional file 4: Fig. S4B). Due to the global loss of H3K27me3 at bivalent promoters, most of the bivalent genes were up-regulated in *Eed*^*−/−*^ and *Suz12*^*−/−*^ mESCs. Considering the expression-enhancing role of H3K4me1 bimodal pattern at promoters [28, 29], the increase of the expression of genes in bimodal-loss group was significantly less than those in bimodal-gain group (*p* value < 0.0001, Wilcoxon rank-sum test; Fig. 4C and Additional file 4: Fig. S4C).

Next, 1,112 up-regulated bivalent genes and 227 down-regulated bivalent genes were identified in *Eed*^−/−^ OG2 mESCs (Fig. 4D). As expected, down-regulated bivalent genes were significantly more involved in the bimodal-loss group (*p* value = 1.75e-09, *﻿χ*^*2*^ test; Fig. 4E; Additional file 4: Fig. S4D). There were also up-regulated bivalent genes in the bimodal-loss group, and they lose less H3K4me1 bimodal patterns at their CGI shores compared to the down-regulated bivalent genes, while change of the H3K4me1 at their CGIs was comparable between the two groups (Fig. 4F). Next, we sought to explore the biological significance of those bivalent genes affected by the loss of the bimodal pattern of H3K4me1. ﻿Functional annotation of those up-regulated bivalent genes showed enrichment in meso-endoderm fate determination related pathways such as embryonic organ morphogenesis, mesenchyme development and skeletal system morphogenesis, while down-regulated bivalent genes were involved in neural ectoderm-related pathways such as synapse organization, dendrite development and morphogenesis (Fig. 4G). This alteration of gene signature leads to spontaneous meso-endoderm specification in primed *Eed*^−/−^ and *Suz12*^−/−^OG2 mESCs [44]. Consistently, PRC2 deficiency in hESCs ﻿also causes pluripotency loss and ﻿spontaneous differentiation towards a meso-endoderm fate at the cost of ectodermal specification [44]. To further validate this observation in our study, retinoic acid (RA) was used to treat the ES cells to induce neuroectodermal differentiation. Upon RA treatment, the WT mESCs efficiently differentiated towards neural ectoderm, and expressed known neural lineage markers, such as *Sox1*, *Pax6* and *Nestin*, while the *Eed*^*−/−*^ and *Suz12*^*−/−*^ mESCs ﻿showed compromised differentiation into neural ectoderm as indicated by lack of neurite synapse outgrowth and significantly lower expression of neural lineage markers (*p* value < 0.0001, *t*-test; Fig. 4H).

In summary, an artificial H3K27me3-H3K4me1 transition model was established in mESCs by knockout *Eed* or *Suz12*. Furthermore, this artificial transition upregulated meso-endoderm specific genes and downregulated ectoderm specific genes, which leads to the impaired ectoderm-differentiation of *Eed*^*−/−*^ and *Suz12*^*−/−*^ mESCs by RA treatment. Taken all, these data demonstrate again that the H3K27me3-H3K4me1 transition determines the cell fate specification in the process of stem cell differentiation.

### LSD1 interacts with core members of PRC2 and plays a significant role in the artificial H3K27me3-H3K4me1 transition in mESCs

Since deficiency of PRC2 which catalyzes H3K27 trimethylation disturbs the regular H3K27me3-H3K4me1 transition as shown above, we next explored other histone modifiers that may participate in the regulation of H3K27me3-H3K4me1 transition. ChIP-seq data of MLL2 (H3K4me3 methyltransferase) knockdown mESCs or UTX (Ubiquitously transcribed tetratricopeptide repeat, X chromosome, a H3K27me3 demethylase) knockout mESCs were subjected for analysis. In *Mll2* knockdown cells, the level of both H3K27me3 and H3K4me1 are upregulated (Additional file 5: Fig. S5A), while in *Utx*^*−/−*^ cells, the level of both H3K27me3 and H3K4me1 are downregulated (Additional file 5: Fig. S5B). Together, both *Mll2* and *Utx* regulate the H3K27me3 and H3K4me1 concurrently, rather than interfere with the H3K27me3-H3K4me1 transition.

**Fig. 5:**
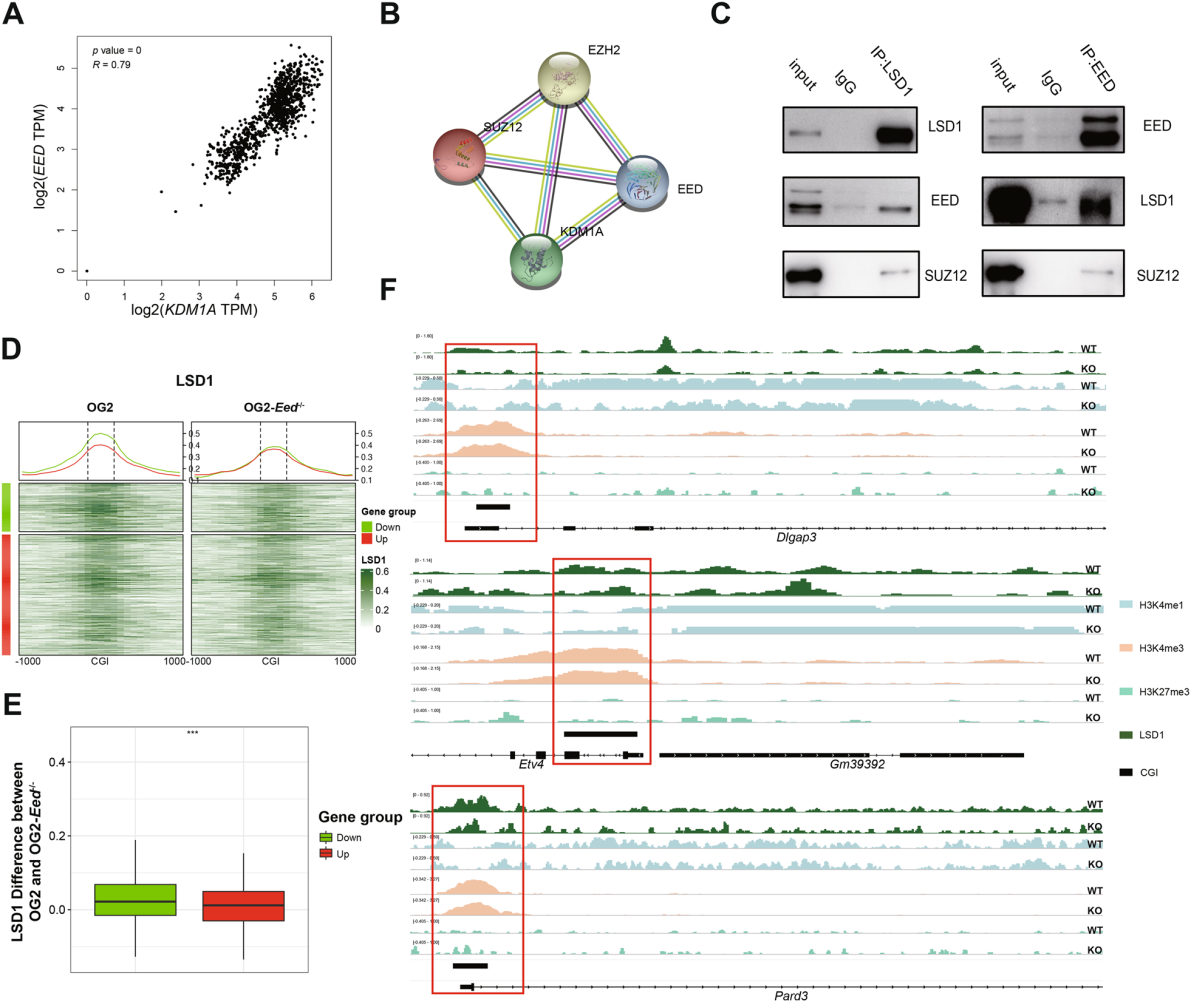
LSD1 interacts with core members of PRC2 and plays a significant role in the artificial H3K27me3-H3K4me1 transition in mESCs. **A** Scatterplot showing the positive correlation of expression between *LSD1* and *EED*. Significance was examined with paired *t*-test, *p* value = 0, *R* = 0.79. **B** The STRING website predicts interaction between LSD1 and SUZ12, EED and EZH2. **C** Left panel: mESC cell nuclear extract was immunoprecipitated by an anti-LSD1 antibody, and subjected to western blotting analysis with anti-EED and anti-SUZ12 antibody; Right panel: mESC cell nuclear extract was immunoprecipitated by an anti-EED antibody, and subjected to western blotting analysis with anti-LSD1 and anti-SUZ12 antibody. **D** Heatmaps and average line plots showing LSD1 patterns at promoter CGIs and their shores of up-regulated (Up) and down-regulated (Down) genes in bimodal-loss group in WT and *Eed*^*−/−*^ mESCs. OG2: WT OG2 mESCs; OG2 *Eed*^*−/−*^: *Eed*^*−/−*^ OG2 mESCs. **E** Boxplot showing the differences of normalized LSD1 coverage at bivalent promoter CGIs between WT and *Eed*^*−/−*^ OG2 mESCs of up-regulated (Up) and down-regulated (Down) genes in bimodal-loss group. Significance was examined *t*-test, ****p value < 0.0001. **F** IGV browser track showing histone modifications patterns and the level of LSD1 chromatin binding at promoter CGIs of neurodevelopmental bivalent genes in WT and *Eed*^*−/−*^ OG2 mESCs

Next, further analysis was performed to dissect the mechanism on how EED and SUZ12 regulate the H3K27me3-H3K4me1 transition. RNA-seq data in multiple tissues in the GTEx project were subjected for expression correlation analysis. LSD1, the H3K4me1 demethylase that binds promoter regions [45, 46], showed strong correlation with EED (Fig. 5A). Moreover, LSD1 was identified to be able to interact EED, SUZ12 and EZH2 based on an online functional protein association network tool STRING (http://string-db.org/) (Fig. 5B). Consistently, previous study has reported that EED, SUZ12 and EZH2 interact with LSD1 in MCF-7 cells [47]. LSD1 also interacts with DEAD-box helicase 19A (DDX19A), which binds H3K27me3 in NIH/3T3 cells [48]. Next, immunoprecipitation was performed and confirmed the interaction between the endogenous EED, SUZ12 and LSD1 in mESCs (Fig. 5C). Taken altogether, these results and previous reports established that LSD1 interacts with EED and SUZ12 across multiple cell types, including mESCs.

Since LSD1 binds promoter regions and interact with EED and SUZ12, we then sought to examine whether LSD1 contributes to the loss of bimodal pattern of H3K4me1 at promoters of bimodal-loss group in *Eed*^*−/−*^ and *Suz12*^*−/−*^ mESCs. ChIP-seq of LSD1 was ﻿performed in wild-type OG2-mESCs, *Eed*^*−/−*^ and *Suz12*^*−/−*^ OG2 mESCs. Notably, ﻿in *Eed*^*−/−*^ and *Suz12*^−/−^ mESCs, LSD1 chromatin binding level was decreased at bivalent promoter CGIs of bimodal-loss group (Fig. 5D, ﻿Additional file 5: Fig. S5C). However, the decrement of LSD1 chromatin binding level at promoter CGIs of down-regulated genes was significantly more than those of up-regulated genes in bimodal-loss group (*p* value < 0.001, *t*-test; Fig. 5E, Additional file 5: Fig. S5D). The more decrement of LSD1 chromatin binding level at promoter CGIs of down-regulated genes involved in bimodal-loss group results in its inability to exert demethylation of H3K4me1, leading to the more loss of bimodal pattern of H3K4me1 which further represses the expression of bivalent genes regulating neural ectoderm development, such as *Dlgap3*, *Pard3* and *Etv4* (Fig. 5F). The Discs large associated protein 3 (*Dlgap3*) was observed throughout the mouse brain [49], that acts as scaffold proteins in the postsynaptic density to control the downstream signaling [50]. Partitioning defective 3 homolog (*PARD3*) is a gene implicated in later aspects of neural tube development [51, 52] and its mutations in human associated with increased risk of neural tube defects [53]. The ETS transcription factors of the PEA3 group, including ETV1, ETV4, and ETV5, are involved in critical physiological processes, such as ectoderm development, organogenesis, and morphogenesis [54]. Consistent with lack of neurite synapse outgrowth and significantly lower expression of neural lineage markers in *Eed*^*−/−*^ and *Suz12*^*−/−*^ mESCs upon RA treatment (Fig. 4H), embryoid bodies derived from *Etv4/5* dKO mESCs did not express ectoderm marker genes [55]. Moreover, downregulation of Etv4 and Etv5 inhibited nerve growth factor (NGF) induced neurite outgrowth of rat sensory neurons [56]. To this end, our data suggested that the loss of the H3K4me1 bimodal pattern is mediated by the decrement of LSD1 chromatin binding level at bivalent promoter CGIs.

Next, to further support these results, *Lsd1* knock-out mESCs (*Lsd1*^*−/−*^ mESCs) were generated through CRISPR-Cas9 (Additional file 6: Fig. S6A). ChIP-seq of histone modifications and RNA-seq were performed subsequently on the *Lsd1*^*−/−*^ mESCs. According to the changes of distribution and level of H3K4me1, all the bivalent genes are divided into two groups, named as L-bimodal-loss group and L-bimodal-gain group, respectively (Additional file 6: Fig. S6B). Surprisingly, *Lsd1* knock-out resulted in a significantly decreased level of H3K27me3 at promoter CGIs of both groups (Additional file 6: Fig. S6B), which led to the genes that belong to the L-bimodal-loss group in *Lsd1*^*−/−*^ mESCs undergoing a similar artificial H3K27me3-H3K4me1 transition observed in *Eed*^*−/−*^ mESCs (Additional file 6: Fig. S6B). Consistently, there were considerable overlaps of genes between L-bimodal-loss group in *Lsd1*^*−/−*^ mESCs and bimodal-loss group in *Eed*^*−/−*^ mESCs: 61% of the genes in L-bimodal-loss group were also included in bimodal-loss group in *Eed*^*−/−*^ mESCs (Additional file 6: Fig. S6C). Like previous observation in *Eed*^*−/−*^ mESCs, the increase of the expression of genes in L-bimodal-loss group was significantly less than that in L-bimodal-gain group (*p* value < 0.0001, Wilcoxon rank-sum test; Additional file 6: Fig. S6D). Moreover, there are more down-regulated genes in the L-bimodal-loss group compared with L-bimodal-gain group (*p* value = 4.438e-14, *﻿χ*^*2*^ test; Additional file 6: Fig. S6E). Next, to examine whether *Lsd1* deficient mESCs also showed impaired neuroectodermal differentiation, we used RA to induce neuroectodermal differentiation. We found that WT mESCs exhibited loss of pluripotency with lost expression of *GFP* driven by *Oct4* promoter as well as neurite synapse outgrowth after 48 h with RA treatment (Additional file 6: Fig. S6F). However, *Lsd1*^*−/−*^ cells show a large population of dead cells after 48 h with RA treatment, and a very small fraction of the survival cells maintained pluripotency with stable expression of *GFP* regulated by *Oct4* promoter, confirming the previous finding that LSD1 is required for neural ectoderm development [57–60] (Additional file 6: Fig. S6F).

**Fig. 6:**
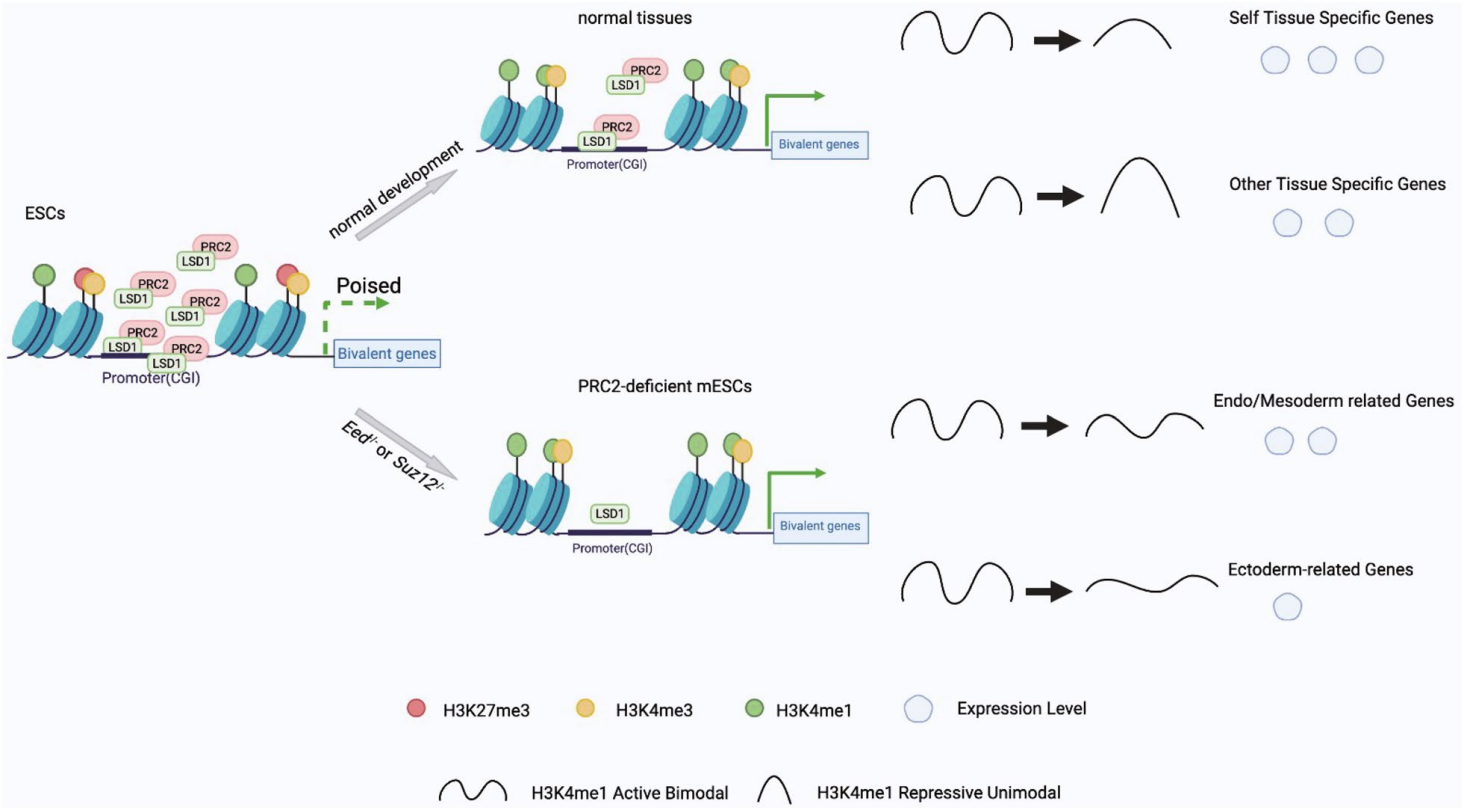
Model for the H3K27me3-H3K4me1 transition in normal developmental process and PRC2-dificient mESCs. Top: During normal tissue/cell development, the H3K27me3-H3K4me1 transition regulates linage specification through the lower level of H3K4me1 unimodal reserves higher transcription level of self-tissue specific bivalent genes compared with other tissue-specific genes during development. Bottom: In *Eed*^*−/−*^ or Suz12^−/−^ mESCs, an artificial H3K27me3-H3K4me1 transition model: loss of H3K27me3 with loss of bimodal pattern of H3K4me1, was established. In this modal, genes related meso-endoderm differentiation acting as self-tissue specific genes were up-regulated owing to more loss of bimodal pattern of H3K4me1 and less decrement of LSD1 chromatin binding level at CGIs, while genes related ectoderm-differentiation acting as other tissue-specific genes were down-regulated owing to less loss of bimodal pattern of H3K4me1 and more decrement of LSD1 chromatin binding level at CGIs

In summary, these results suggested that LSD1 interacts with PRC2 in mESCs and mediates the alteration of H3K4me1 bimodal pattern at bivalent promoter CGIs, which orchestrates the expression of a subset of genes that regulates the neural ectoderm development in the early developmental stage of mESCs (Fig. 6).

## Discussion

Bivalent genes, initially identified in ESCs, maintain a transcriptionally poised state in ESCs, and are able to be activated upon appropriate activation cues during development [1, 5–9]. In current study, we reported that H3K4me1 in combination with H3K4me3 considerably distinguish bivalent and non-bivalent promoter CGIs in ESCs. Most traditional bivalent promoters are trivalent promoters, co-labelled by H3K4me1, H3K4me3 and H3K27me3 in ESCs. Next, we found that these bivalent promoter CGIs undergo the H3K27me3-H3K4me1 transition, and this transition regulates tissue-specific genes expression during lineage differentiation. Finally, LSD1 could modulate ﻿the H3K27me3-H3K4me1 transition via interfering with PRC2. Taken altogether, our results revealed that H3K27me3-H3K4me1 transition and the pattern of H3K4me1 imposed by the transition orchestrates the tissue specification in development. This study provided more insights into understanding the role of H3K4me1 modification at bivalent promoters during cell fate decision and lineage specification at early stages of development (Fig. 6).

Firstly, we reported that H3K4me1 in combination with H3K4me3 represent most traditional bivalent promoters in hESCs and mESCs, which means that, to some extent, H3K4me1 represents H3K27me3 at bivalent promoters. Furthermore, we found an untypical H3K4me1 bimodal pattern at bivalent promoters, while active promoters showed a typical H3K4me1 bimodal pattern. Consistently, previous studies have shown two different distribution patterns of H3K4me1 at promoters and the co-occupancy of H3K4me1 and H3K27me3 at promoters in various cell types [28, 29, 31, 32]. Furthermore, in germs cells and ES cells, unimodal H3K4me1 patterns correlate strongly with poised promoters marked by both H3K4me3 and H3K27me3 [29]. Collectively, these results show that most traditional bivalent promoters are trivalent promoters, co-labelled by H3K4me1, H3K4me3 and H3K27me3 in ESCs, and H3K4me1 plays a significant role in regulating bivalent promoters.

Previous studies have shown that there is regulatory crosstalk between H3K4 methylation and H3K27me3. The dynamics of these histone modifications orchestrates the expression of genes. The catalytic activity of PRC2 was greatly reduced on the histone tail which is already modified by H3K4 methylation [61], and thus H3K27me3 modification usually happens on the other H3 tail within the same nucleosome, allowing the formation of bivalent domains [62]. Consistently, bivalent promoters showed a negative correlation between H3K4me3 and H3K27me3. In *Mll2*^*−/−*^ mESCs, the loss of H3K4me3 is accompanied by gain of H3K27me3 on the bivalent promoters, which caused downregulation of these genes [63, 64]. A similar result was also observed at the promoter of indicated PRC2-targeted genes such as *Wnt5* during mESCs differentiation induced by RA [65]. However, ESCs depleted of *Rbbp5* or *Dyp-30* (Dumpy-30), ﻿two core components that were ﻿integral and shared components of all the COMPASS family complexes, shows decreased H3K4me3 and a marginal decreased H3K27me3 on the promoter of multiple ESC specific genes [66]. Another study in NIH/3T3 cells showed that the induced binding of LSD1 to a synthetic promoter resulted in increased H3K27me3 with decreased H3K4me2, which inhibited expression of *mCherry* driven by this synthetic promoter [48]. In current study, we reported a novel general phenomenon, the H3K27me3-H3K4me1 transition at bivalent promoter CGIs during development. The loss of H3K27me3 was accompanied by a bimodal pattern loss or unimodal pattern enrichment of H3K4me1 during development in multiple tissues in both human and mouse. Previously, most of the enhancers identified by the enrichment of H3K4me1 modification are functionally active in some types of cells [67]. Remarkably, however, in this study, H3K4me1 acts as a context-dependent conditional repressor at bivalent promoter CGIs and regulates the expression of tissue-specific genes. Further analysis revealed that this transition promotes embryonic development through lower enrichment of unimodal H3K4me1 at the tissue-specific bivalent promoter CGIs compared with other bivalent genes in each specific tissue.

Poised enhancers, marked by both positive (H3K4me1) and repressive (H3K27me3) histone modifications, are another class of bivalent regions[2]. The presence of H3K27ac on enhancers distinguishes active enhancers from poised enhancers [23], implicating there may be a potential transition from H3K27me3 to H3K27ac. Moreover, during embryo development, the loss of H3K27me3 and gain of H3K27ac at poised enhancers prepare the poised enhancers for future activation [68], also indicating the H3K27me3-H3K27ac transition. Based on these observations, it is plausible that a histone modification transition exists at poised regions, including both poised enhancers and promoters. However, further studies are necessary to explore mechanism of poised enhancers regulating the expression of tissue-specific genes.

Core members of PRC2 complex are essential for embryonic development [10, 69, 70]. PRC2 complex represses gene expression by mediating histone modifications such as H3K27me3 deposition or through other epigenetic mechanisms [71, 72]. Mutations or inactivation in core components such as *Eed*, *Suz12* and *Ezh2* in mice result in early lethality [14]. Previous study has shown that *EZH1* or *EZH2* null H1 cells (hESCs) spontaneously differentiate towards the meso-endoderm at the cost of neural ectoderm differentiation [44]. Additionally, *Suz12*^*−/−*^ mESCs were unable to form neurons [73]. Loss of *Eed* in the mouse brain leads to postnatal lethality, impaired neuronal differentiation, and malformation of the dentate gyrus [74]. These results suggested that PRC2 components play indispensable roles in deciding neural ectoderm fate, but the detailed molecular mechanisms underlying neural ectoderm specification defects in PRC2-deficient mESCs remain unclear. In current study, an artificial H3K27me3-H3K4me1 transition model: loss of H3K27me3 with loss of bimodal pattern of H3K4me1, was established in mESCs by knockout *Eed* or *Suz12*. In this modal, genes related to meso-endoderm differentiation acting as self-tissue specific genes were up-regulated while genes related to ectoderm-differentiation acting as other tissue-specific genes were down-regulated owing to this new artificial transition, which probably further lead to spontaneous meso-endoderm differentiation in prime *Eed*^*−/−*^ and *Suz12*^*−/−*^ mESCs [44], and ectoderm-differentiation failure upon the induction by RA treatment. Taken together, we provided additional evidences to explain how neural ectoderm development is impaired in *Eed*^*−/−*^ and *Suz12*^*−/−*^ mESCs through epigenetic mechanisms.

In addition, we found that LSD1 plays a critical role in this transition process. In this study, consistent with previous reports, we demonstrated the interaction of LSD1 with EED and SUZ12 in mESCs [47, 48]. This interaction stabilizes the binding of LSD1 to the promoter region of target genes. So, in *Eed*^*−/−*^ and *Suz12*^*−/−*^ mESCs, the genes undergoing an artificial transition owing to the decrement of LSD1 chromatin binding level at their promoters. Similarly, in *Lsd1*^*−/−*^ mESCs, an artificial H3K27me3-H3K4me1 transition also was observed at promoters of L-bimodal-loss bivalent genes. These findings are consistent with previous studies demonstrating that LSD1 is necessary for neural ectoderm development. In cortical progenitors, LSD1 forms a complex with the transcriptional corepressor CoREST, that opposes Notch signaling pathway to promote cortical neuronal differentiation [57]. Similar functions for LSD1 were observed in human fetal neural stem cells (hfNSCs), wherein LSD1 controls the demethylation of H3K4me2 at the promoter of the HEYL gene to regulate the neuronal differentiation of hfNSCs [57]. And loss of LSD1 in the mouse adult brain resulted in widespread neuronal death throughout the cortex and hippocampus [75]. In addition, LSD1 has a neuronal-specific splice variant (referred to as LSD1n, neuronal form), that is required for neuronal maturation [59, 60, 75]. However, our results are still limited and more detailed molecular mechanisms underlying neural ectoderm differentiation defects in *Eed*^−/−^, *Suz12*^−/−^ and *Lsd1*^*−/−*^ mESCs are required.

## Conclusion

Our report defines a novel role for H3K4me1 at bivalent promoters, as a conditional repressor to regulate tissue-specific gene expression during development. More importantly, we propose that LSD1-EED/SUZ12 axis, influences early cell fate determination through regulating the H3K27me3-H3K4me1 transition in mESCs. In summary, we provide more evidences to explain epigenetically how neural ectoderm differentiation is impaired in *Eed*^−/−^ and *Suz12*^−/−^ mESCs. Furthermore, our results provided more insights into the understanding of cell fate decisions mediated by PRC2 complex and LSD1 via regulating histone modifications of bivalent promoters during lineage specification.

## Materials and methods

### Cell culture

All the WT, *Eed*^*−/−*^ and *Suz12*^*−/−*^ OG2 cell lines were kindly provided by Professor Guangjin Pan in Guangzhou Institutes of Biomedicine and Health, Chinese Academy of Sciences. These mouse ESCs were maintained on gelatin-coated plate in DMEM/ high glucose (Gibco, C11995500BT) supplemented with 15% FBS (Lonsera, S712-012S), NEAA (Gibco, 11140050), GlutaMAX (Gibco, 35050061), Sodium Pyruvate (Gibco, 11360070), 1 μM PD0325901 (TargetMol, T6189-1 mL), 3 μM CHIR99021 (TargetMol, T2310-2 mg), 100 μM β-mercaptoethanol (MERCK, M6250), 1000 units/mL mLIF (Millipore, ESG1107). All cells were maintained at 37 °C, 5% CO2.

### ESC differentiation

For neural ectoderm differentiation, mESCs were adapted on gelatin-coated plate in N2B27 medium (50% DMEM/F12 (Hyclone, SH30023.01), 50% Neurobasal (Gibco, 21103049), N2 (Gibco, 17502048), B27 (Gibco, 17504044), NEAA (Gibco, 11140050), GlutaMAX (Gibco, 35050061), Sodium Pyruvate (Gibco, 11360070), 100 μM β-mercaptoethanol (MERCK, M6250)) + 2iL (1 μM PD0325901 (TargetMol, T6189-1 mL), 3 μM CHIR99021 (TargetMol, T2310-2 mg), 1000 units/mL mLIF (Millipore, ESG1107)) for a minimum of 4 days, and then the cells were seeded on gelatin-coated 6-well plate in N2B27 medium for 48 h. Afterwards, cells were treated with 500 nM retinoic acid (RA) in N2B27 medium for 4 days before subjected for subsequent analysis. Medium was changed every 2 days.

### Generation of CRISPR knockout clones

The sequence CCTGAGAGGTCATTCGGTCA in exon 3 of LSD1 was selected as the CRISPR target as previously described [76] and subcloned into the CRISPR/Cas9 gene editing vector pSpCas9(BB)-2A-Puro (PX459) V2.0 (Addgene, #62988), a gift kindly provided by Professor Junjun Ding in Zhongshan school of medicine, Sun Yat-sen University. OG2 mESCs were transiently transfected with CRISPR/Cas9 vector via Lipofectamine3000 (Thermo Fisher, L3000015) and then subjected to puromycin (1 μg/mL) (Shanghai yuanye Bio-Technology, R23002) treatment for 24 h for selection. Individual clones were further expanded. The depletion of LSD1 was confirmed by immunoblotting.

### Western blot analysis

The whole cell extracts were prepared by RIPA buffer (Solarbio, RIPA-56) and then subjected to SDS–polyacrylamide gel electrophoresis (PAGE). Proteins in the gel were then transferred to PVDF membranes (Millipore, ISEQ00010), and incubated with primary antibodies over-night at 4 °C. HRP-conjugated secondary antibodies were used and bands were visualized with the enhanced chemiluminescence detection (ECL) (ECOTOP, 5008-B). All uncropped western blots can be found in Additional file 7: Fig. S7. The information for antibodies used is listed in Additional file 8: Table S1.

### Quantitative real-time PCR

Total RNA was extracted with Total RNA Kit I (Omega Bio-Tek, R6834-02), and reverse transcribed with RevertAid First Strand cDNA Synthesis Kit (Thermo Fisher Scientific, K1622), and then Quantitative real-time PCR qPCR was performed with ChamQ Universal SYBR qPCR Master Mix (Vazyme, Q711-02) following the manufacturer’s recommendations. *Gapdh* was used for qRT-PCR normalization of mouse sample. For statistical analysis of this experiments, all the data were measured in three repeats and results are presented as mean ± SD and tested with *t*-test (GraphPad Prism software) to calculate the *p* values between unpaired samples. All primer sequences are listed in Additional file 9 Table S2.

### Co-immunoprecipitation assay

mESC cells were lysed in hypotonic lysis buffer (10 mM HEPES, pH 7.9, 10 mM KCl, 0.1 mM EDTA, 0.1 mM EGTA, and cOmplete protease inhibitors (Roche, 4693116001)) and incubated on ice for 15 min. The lysates were centrifuged for 10 min at 1,530 g and the supernatant (cytoplasmic extract) was discarded. The pelleted nuclei were washed once with hypotonic lysis buffer and then resuspended in hypertonic buffer (20 mM HEPES, pH 7.9, 0.4 M NaCl, 1 mM EDTA, 1 mM EGTA, 0.6% NP-40 and cOmplete protease inhibitors (Roche, 4693116001)) with 100U/mL DNase I (NEB, M0303S) for 45 min at 4 °C, and spun down at 13,800 g for 10 min at 4 °C. The supernatant (nuclear extracts) was diluted twofold with IP buffer (20 mM HEPES, pH 7.9, 0.2 M NaCl, cOmplete protease inhibitors (Roche, 4693116001) and then precleared by the Protein-G magnetic beads (Thermo Fisher Scientific, 01134323) for 1 h with rotation at 4 °C. Then the supernatant was incubated with IgG (abcam, ab150157) or specific antibodies (EED ab240650, abcam), LSD1 (2184, cell signaling technology)) overnight with rotation at 4 °C, followed by incubation with Protein-G magnetic beads for 2 h with rotation at 4 °C. The immune-complex were then washed with IP buffer (20 mM HEPES, pH 7.9, 0.2 M NaCl, 0.3% NP-40, cOmplete protease inhibitors (Roche)) for five times. Bound proteins were then eluted in sample buffer (62.5 mM Tris, pH 6.8, 10% Glycerol, 2% SDS, 5% beta-mercaptoethanol, and bromophenol blue) and subjected to western blot analyses. The information for antibodies used is listed in Additional file 8: Table S1.

### Chromatin immunoprecipitation (ChIP)

For H3K4me1-, H3K4me3- and H3K27me3-ChIP experiments, 5 × 10^6^ cells were cross-linked with 1% formaldehyde in 10 mL PBS for 10 min at room temperature. The reaction was stopped by the addition of glycine (125 mM). After washing with PBS, cells were lysed and then rotated in Lysis Buffer 1 (50 mM HEPEDS-KOH pH 7.5, 1 mM EDTA, 140 mM NaCl, 10% Glycerol, 0.5% NP-40, 0.25% Triton X-100 and cOmplete protease inhibitors (Roche, 4693116001)) for 10 min at 4 °C and then spun down at 1150 g for 5 min at 4 °C. The cells were then resuspended and rotated in Lysis Buffer 2 (10 mM Tris–HCl pH8.0, 200 mM NaCl, 1 mM EDTA, 0.5 mM EGTA and cOmplete protease inhibitors (Roche)) for 10 min at RT and spun down at 1150 g for 5 min at 4 °C.

After lysis, cells were sonicated in shearing buffer (10 mM Tris–HCl pH8.0, 1 mM EDTA, 0.1% SDS) to shear DNA to lengths between 200 and 1000 base pairs by Covaris M220 with 5% duty factor for 10 min at 4 °C. The sonicated samples were added 1% Triton X-100 and 150 mM NaCl and then were diluted with shearing buffer to 1 ml and spun down at 20,000 g for 10 min at 4 °C. The supernatant was precleared by the Protein-G magnetic beads (Thermo Fisher Scientific, 01134323) for 1 h with rotation at 4 °C and then incubated with IgG (abcam, ab150157) or specific antibodies (H3K4me1 (abcam, ab8895), H3K4me3 (abcam, ab8580), H3K27me3 (Active Motif, 39,055)) overnight with rotation at 4 °C, followed by incubation with Protein-G magnetic beads for 2 h with rotation at 4 °C. The immune-complex were then washed with IP buffer (10 mM Tris–Cl, pH 8.0, 150 m M NaCl, 1% Triton X-100, 1 mM EDTA, 0.1% SDS), High Salt Wash Buffer (20 mM Tris–Cl, pH 8.0, 500 m M NaCl, 1% Triton X-100, 2 mM EDTA, 0.1% SDS), LiCl Wash Buffer (10 mM Tris–Cl, pH 8.0, 250 mM LiCl, 2 mM EDTA, 1% NP-40) and TE + 50 mM NaCl Buffer for two times at 4 °C. The immune complex were then eluted with elution buffer (10 mM Tris–Cl, pH 8.0, 10 mM EDTA, 1% SDS) by shaking at 1,400 rpm at 65 °C for 30 min. Supernatant was de-crosslinked in elution buffer by incubating at 65 °C for 16 h, and then treated with 20 mg/mL RNaseA at 37 °C for 2 h and 20 mg/mL Proteinase K (MIKX, FZ690) at 55 °C for 2 h. DNA was purified by phenol–chloroform-isoamyl alcohol (Solarbio, P1012-100) and ethanol precipitation, and subjected for further analysis including RT-PCR and ChIP-seq. All primer sequences are listed in Additional file 9: Table S2.

For LSD1-ChIP, 5 × 10^6^ mESC cells were fixed and harvested as described above. 3ug LSD1 antibody (ab17721, abcam) was used for each IP assay in weak IP buffer (10 mM Tris–Cl, pH 8.0, 150 m M NaCl, 0.1% Triton X-100, 1 mM EDTA, 0.1% SDS) followed by incubation with Protein-G magnetic beads for 2 h with rotation at 4 °C. The immune-complex were then washed four times with weak IP buffer and then once with TE buffer before elution.

### Data source

The ChIP-seq data of H3K4me1, H3K4me3, H3K27me3, as well as RNA-seq data for WT, *Eed*^*−/−*^, *Suz12*^*−/−*^, *Lsd1*^*−/−*^ mESCs and LSD1 ChIP-seq data for WT, *Eed*^*−/−*^, *Suz12*^*−/−*^ mESCs generated during our study are available at Gene Expression Omnibus: GSE217249. Public sequencing data used in this study were obtained from multiple sources: GEO dataset (http://www.ncbi.nlm.nih.gov/geo), Encode dataset (https://www.encodeproject.org/), Roadmap (https://egg2.wustl.edu/roadmap/web_portal/processed_data.html). See Additional file 10 Table S3 for details.

### ChIP-seq data analysis

FastQC (version 0.11.9) was used to access the base quality of raw data and trim_galore (version 0.0.1) was used to trim the adaptor and low-quality reads with parameters -q 25 -phred 33 -length 40 −e 0.1 -stringency 3. After quality control, the remaining reads were mapped to the reference genome (mm10) with bowtie2 (version 2.4.5). Only uniquely mapped reads were kept and duplicates were removed by sambamba (version 0.8.2). Peaks were called using MACS2 (version 2.2.7.1) with the significance cut-off *q*-value ≤ 0.05. Bigwig files adjusted by input data and enrichment profiles across genomic regions of interest were all generated using deeptools (version 3.5.1). Filtered bam files of Encode human and mouse histone data were download and converted to bigwig files which adjusted by input data using deeptools (version 3.5.1).

### Histone modification distribution analysis

Promoter CpG islands were defined as CGIs (200–5000 bp) which overlapped with TSS ± 1 kb regions. H3K4me1, H3K4me3, H3K27me3 histone signals were normalized to these promoter CGIs with extended upstream and downstream 1 kb regions by R package ‘EnrichedHeatmap’ (version 1.24.0). *K*-means clustering algorithm was used to reveal three major groups of promoter CGIs based on the all three histone modifications in human and mouse ESCs. Signals of other ChIP-seq data and DNA methylation data were then normalized to these grouped promoter CGIs for incorporate analysis. For *Eed/Lsd1* WT/KO histone data, *k*-means clustering algorithm was performed based on H3K4me1 histone signals in CpG islands and its upstream and downstream 1 kb regions respectively. Then by combining the groups clustered based on these two types of regions, the final two H3K4me1 unimodal and bimodal inter conversional groups were defined.

### Gene expression analysis

FastQC (version 0.11.9) was used to access the base quality of raw data and trim_galore (version 0.0.1) was used to trim the adaptor and low-quality reads with parameters -q 25 -phred 33-length 40 -e 0.1 -stringency 3. After quality control, the remaining reads were mapped to the reference genome (hg19, mm10) with hisat2 (version 2.2.1). Reads mapping to multiple locations were filtered out. Read count extraction and normalization were performed using HTSeq (version 2.0.2). Differential gene expression analysis was performed using R package ‘DESeq2’ (version 1.34.0). For human ESC and differentiated tissues, differentially expression genes were defined with the certain threshold: ± 2 log2FC (*p* value ≤ 0.05). While genes that displayed ± 1.2 log2FC (*p* value ≤ 0.1) between WT and *Eed/Lsd1* KO mouse ESC cells were considered as significantly differentially expressed.

### Function enrichment analysis

Function enrichment analysis was performed by R package ‘clusterProfiler’ (version 4.2.2). GO term and Reactome Pathway enrichment were calculated. Adjusted *p* value < 0.05 were used as significant cutoff. All CpG islands related genes or genes captured by RNA-seq were selected as universal genes.

### Tissue-specific gene and tissue enrichment analysis

Tissue-specific genes were selected as previous paper [38] (Tissue-specific transcription reprogramming promotes liver metastasis of colorectal cancer). For each gene, we ranked the median expression value for each tissue in decreasing order. Genes defined as tissue-specific needed to meet two criteria: (1) gene expression ranked in the top 5 among all tissue; (2) also highly expressed (> 90th percentile of all genes) tissues. Tissue enrichment analysis was conducted using R package ‘TissueEnrich’ (version 1.14.0).

### Logistic regression analysis

Logistic regression analysis was used to test the contribution of histone modifications in CpG islands and enhancer regions of corresponding genes to tissue specificity. Enhancers was defined using H3K4me1 peaks 5 kb away from TSS. To obtain histone signal differences between corresponding genes in ESC and differentiated tissues, only the nearest enhancer was retained for each gene. Logistic regression analysis was conducted to estimate the relationship between the dependent variable (whether it was a tissue-specific gene) and independent variables (H3K4me1, H3K4me3 and H3K27me3 histone signals in CpG islands and H3K4me1, H3K27ac histone signals in enhancer regions). R packages ‘caret’ (version 6.0) and ‘epiDisplay’ (version 3.5.0.2) were used for this process and R package ‘forestplot’ (version 2.0.1) was used to plot forest plot.

### Statistical analysis

All statistical analysis were performed by R (version 4.1.3). Tests involving comparisons among multi-groups were performed using Kruskal Wallis rank-sum test or ANOVA, comparisons between two-groups were performed using Wilcoxon rank-sum test or *t*-test. Tests involving comparisons of number or ratio were performed using *χ*^*2*^ test, ***p value < 0.001, **p value < 0.01, *p value < 0.05; ns, not significantly, *p* value > 0.05.

Bioinformatics tool URLs:

R: https://www.r-project.org/

Bowtie: http://bowtie-bio.sourceforge.net/index.shtml

SRA ToolKit: https://github.com/ncbi/sra-tools

fastQC: http://www.bioinformatics.babraham.ac.uk/projects/fastqc/

Sambamba: https://lomereiter.github.io/sambamba/

SAMTools: http://www.htslib.org/

BEDTools: http://bedtools.readthedocs.org/en/latest/

deepTools: http://deeptools.readthedocs.org/en/latest/

MACs tools: https://github.com/taoliu/MACS

HITSAT2: http://daehwankimlab.github.io/hisat2/

HTSeq: https://htseq.readthedocs.io/en/master/

IGV Tools: https://www.broadinstitute.org/igv/igvtools

IGV browser: https://www.broadinstitute.org/igv/

UCSC browser: http://genome.ucsc.edu/

## Supplementary Information

**Additional file 1: Figure S1.** H3K4me1 in combination with H3K4me3 is able to partition promoters and predict bivalent promoters. **A** Heatmaps showing histone modifications patterns of promoter CGIs and their shores based on the distribution patterns of traditional bivalent marks (H3K4me3 and H3K27me3) (left) or non-traditional bivalent marks (H3K4me1 and H3K4me3) (right), respectively in hESCs; Sankey Diagram showing considerable overlaps between the clusters defined based on the two different bivalent marks combinations described above. **B** Heatmaps and average line plots showing histone modifications patterns and average methylation patterns of promoter CGIs and their shores in mESCs. Each line represents a single CpG island. Right panel: Heatmaps and boxplots showing gene expression and tissue-specific score (Tau) of three different clusters in mESC. **C** Histograms showing GO-term enrichment for genes involved in three clusters as in (B) in mESCs. **D** Heatmaps and average line plots showing H3K4 and H3K27 methylation related HMTs (EZH2, SZU12 for H3K27; KMT2B, KMT2C, KMT2D for H3K4; RYBP, CBX8 for H2AK119ub) patterns of promoter CGIs and their shores of three clusters as in (B) in mESCs, respectively. Each line represents a single CpG island.

**Additional file 2: Figure S2.** Bivalent promoter CGIs undergo H3K27me3-H3K4me1 transition during development. **A** Heatmaps showing H3K4me1, H3K4me3 and H3K27me3 patterns of promoter CGIs and their shores in bivalent cluster in hESC, normal human lung fibroblasts (NHLF) and other human tissues (liver, spleen, stomach, small intestine and pancreas). Each line represents a single CpG island. **B** Heatmaps showing H3K4me1, H3K4me3, H3K27me3 and H3K27ac patterns at enhancers in hESC and lung. Each line represents a single enhancer. **C** Heatmaps showing H3K4me1, H3K4me3 and H3K27me3 patterns of promoter CGIs and their shores in bivalent cluster in mESC and mouse tissues (liver, kidney, spleen and cerebellum). Each line represents a single CpG island. **D** Heatmaps showing H3K4me1, H3K4me3 and H3K27me3 patterns of promoter CGIs and their shores in bivalent cluster between mouse fibroblasts (MEF) and their reprogrammed iPSC cells. Each line represents a single CpG island.

**Additional file 3: Figure S3.** H3K27me3-H3K4me1 transition influences tissue-specific gene expression. **A** Top panel: boxplots showing the differences of H3K4me1, H3K4me3 and H3K27me3 on promoters of tissue specific genes between hESC and liver. All Other: all the other tissues without liver. Bottom panel: boxplots showing the differences of H3K4me1, H3K4me3 and H3K27me3 on promoters of tissue specific genes between hESC and pancreas. All Other: all the other tissues without pancreas. Lung: tissue-specific bivalent genes of lung; Liver: tissue-specific bivalent genes of liver; Pancreas: tissue-specific bivalent genes of pancreas; Spleen: tissue-specific bivalent genes of spleen. Significance was examined with *t*-test. *p value < 0.05, **p value < 0.01, ****p value < 0.0001﻿, ns: not significantly. **B** Top panel: boxplots showing levels of H3K4me1 and H3K27ac of tissue-specific bivalent genes at enhancers in liver. All Other: all the other tissues without liver. Bottom: boxplots showing levels of H3K4me1 and H3K27ac of tissue-specific bivalent genes at enhancers in pancreas. All Other: all the other tissues without pancreas. Lung: tissue-specific bivalent genes of lung; Liver: tissue-specific bivalent genes of liver; Pancreas: tissue-specific bivalent genes of pancreas; Spleen: tissue-specific bivalent genes of spleen. Significance was examined with *t*-test. *p value < 0.05, ****p value < 0.0001﻿, ns: not significantly. **C** Boxplot showing interaction score between promoter and enhancer of liver or spleen tissue-specific and all other (liver or spleen not included) tissue-specific bivalent genes in liver or spleen (Significance was examined with Wilcoxon rank-sum test, ****p value < 0.0001﻿).

**Additional file 4: Figure S4.** An artificial H3K27me3-H3K4me1 transition regulates the ESCs differentiation. **A** Western blotting analysis of H3K27me3 modification in WT and *Suz12*^*−/−*^ OG2 mESCs. H3 as the loading control; heatmap showing H3K27me3 patterns of promoter CGIs and their shores in WT, *Eed*^*−/−*^ (replicate) and *SUZ12*^*−/−*^ OG2 mESCs. Each line represents a single CpG island. OG2: WT OG2 mESCs; OG2 *Eed*^*−/−*^: *Eed*^*−/−*^ mESCs; OG2 *Suz12*^*−/−*^: *Suz12*^*−/−*^ mESCs. **B** Heatmaps and average line plots showing H3K4me1, H3K4me3 and H3K27me3 patterns at promoter CGIs and their shores of two different groups (H3H4me1 biloss group and bigain group) in WT, *Eed*^*−/−*^ (replicate) and *SUZ12*^*−/−*^ OG2 mESCs. Each line represents a single CpG island. H3K4me1 Biloss: H3K4me1 bimodal-loss group; H3K4me1 Bigain: H3K4me1 bimodal-gain group. OG2: WT OG2 mESCs; OG2 *Eed*^*−/−*^: *Eed*^*−/−*^ mESCs; OG2 *Suz12*^*−/−*^: *Suz12*^*−/−*^ mESCs. **C** Boxplot showing expression alteration (log2Foldchange) of bivalent genes in H3K4me1 biloss group and H3K4me1 bigain group in *Suz12*^*−/−*^ OG2 mESCs. Significance was examined with Wilcoxon rank-sum test, ****p value < 0.0001. H3K4me1 Biloss: H3K4me1 bimodal-loss group; H3K4me1 Bigain: H3K4me1 bimodal-gain group. **D** Histogram showing the percentage of H3K4me1 biloss group genes and H3K4me1 bigain group genes in the up-regulated (Up) or down-regulated (Down) genes in *Suz12*^*−/−*^ OG2 mESCs compared with WT OG2 mESCs, respectively. Significance level was determined using ﻿*χ2* tests, *p* value ﻿ = 1.159e-07. H3K4me1 Biloss: H3K4me1 bimodal-loss group; H3K4me1 Bigain: H3K4me1 bimodal-gain group.

**Additional file 5: Figure S5.** LSD1 interacts with core members of PRC2 and plays a significant role in the artificial H3K27me3-H3K4me1 transition in mESCs. **A** Heatmaps and ﻿average line plots showing histone modifications patterns of promoter CGIs and their shores in WT, *Mll2*-shRNA and *Mll3*-shRNA mESCs. Each line represents a single CpG island. **B** Heatmaps and ﻿average line plots showing histone modifications patterns of promoter CGIs and their shores in WT and *Utx*^*−/−*^ mESCs. Each line represents a single CpG island. **C** Heatmaps and average line plots showing LSD1 patterns at promoter CGIs and their shores of up-regulated (Up) and down-regulated (Down) genes of bimodal-loss group in WT and *Suz12*^*−/−*^ OG2 mESCs. Each line represents a single CpG island. OG2: WT OG2 mESCs; OG2 *Suz12*^*−/−*^: *Suz12*^*−/−*^ mESCs. **D** Boxplot showing the differences of normalized LSD1 coverage at bivalent promoter CGIs between WT (OG2) and *Suz12*^*−/−*^ (OG2 *Suz12*^*−/−*^) mESCs of up-regulated (Up) and down-regulated (Down) genes in bimodal-loss group. Significance was examined with *t*-test, ****p value < 0.0001.

**Additional file 6: Figure S6.** *Lsd1* knockout induces similar effects as *Eed* or *Suz12* knockout. **A** Western blot analysis of LSD1 protein in each indicated cell lines (OG2, OG2-*Lsd1*^*−/−*^). ACTIN as the loading control. **B** Heatmaps and average line plots showing H3K4me1, H3K4me3 and H3K27me3 patterns at promoter CGIs and their shores of two different groups in WT and *Lsd1*^*−/−*^ OG2 mESCs. Each line represents a single CpG island. H3K4me1 L-Biloss: H3K4me1 L-bimodal-loss group; H3K4me1 L-Bigain: H3K4me1 L-bimodal-gain group. OG2: WT mESCs; OG2 *Lsd1*^*−/−*^: *Lsd1*^*−/−*^ mESCs. **C** Veen diagram showing considerable overlaps of the promoter CGIs undergoing the artificial transition betweent *Lsd1*^*−/−*^ and *Eed*^*−/−*^ mESCs. ﻿*Lsd1* KO Trans CGIs: the promoter CGIs undergoing an artificial transition in *Lsd1*^*−/−*^ mESCs; *Eed* KO Trans CGIs: the promoter CGIs undergoing an artificial transition in *Eed*^*−/−*^ mESCs. **D** Boxplot showing expression alteration (log2Foldchange) of bivalent genes in H3K4me1L- biloss group and H3K4me1 L-bigain group in *Lsd1*^*−/−*^ OG2 mESCs. Significance was examined with Wilcoxon rank-sum test, ****p value < 0.0001. H3K4me1 L-Biloss: H3K4me1 L-bimodal-loss group; H3K4me1 L-Bigain: H3K4me1 L-bimodal-gain group. **E** Histogram showing the percentage of H3K4me1 L-bimodal-loss group genes and H3K4me1 L-bimodal-gain group genes in the up-regulated (Up) or down-regulated (Down) genes in *Lsd1*^*−/−*^ mESCs compared with WT mESCs, respectively. Significance level was determined using *χ2* tests, *p* value ﻿ = 4.438e-14. H3K4me1 L-Biloss: H3K4me1 L-bimodal-loss group; H3K4me1 L-Bigain: H3K4me1 L-bimodal-gain group. **F** Images showing the morphology of mESC cells after RA treatment. WT mESCs is OG2 mESCs with GFP expression controlled by *Oct4* promoter (Oct4: GFP). Scale bar, 100 μm. OG2: WT mESCs; OG2 *Lsd1*^*−/−*^: *Lsd1*^*−/−*^ mESCs.

**Additional file 7: Figure S7.** Uncropped scans of western blots. **A** Uncropped scans of western blots for Fig. 4A and Fig. S4A. Histone modification H3K27me3 level was analyzed by western-blot using the specific antibody on the whole cell lysates from indicated cell lines (OG2, OG2-*Eed*^*−/−*^ and OG2-*Suz12*^*−/−*^). H3 as the loading control. Red box indicates the location of goal protein. *KD* Kilodaltons. **B** Uncropped scans of western blots for Fig. 5C. mESC cell nuclear extract was immunoprecipitated by an anti-LSD1 antibody, and subjected to western blotting analysis with anti-EED (left); mESC cell nuclear extract was immunoprecipitated by an anti-EED antibody, and subjected to western blotting analysis with anti-LSD1 (right). Red box indicates the location of goal protein. **C** Uncropped scans of western blots for Fig. 5C. mESC cell nuclear extract was immunoprecipitated by an anti-LSD1 or anti-EED antibody, and subjected to western blotting analysis with anti-SUZ12. Red box indicates the location of goal protein. **D** Uncropped scans of western blots for Fig. S6A. LSD1 expression level was analyzed in each indicated cell lines (OG2, OG2-*Lsd1*^*−/−*^). ACTIN as the loading control. Red box indicates the location of goal protein.

**Additional file 8: Table S1.** The information for antibodies used in this study.

**Additional file 9: Table S2.** The sequence of all primers used in this study.

**Additional file 10: Table S3.** The information of all public sequencing data used in this study.

## Data Availability

All public sequencing data used in this current study are listed in Additional file 10: Table S3. All data generated during this current study is deposited at NCBI GEO: GSE217249. The materials used during this current study are available from the corresponding author on reasonable request.

## Abbreviations

ESC: Embryonic stem cell
iPSC: Induced pluripotent stem cells
H3K4me3: Trimethylation of histone H3 on lysine 4
H3K27me3: Trimethylation of histone H3 on lysine 27
H3K4me1: Monomethylation on lysine 4 of histone H3
LSD1: Lysine-specific demethylase 1
MLL: Mixed lineage leukemia
EZH2: Enhancer of Zeste homologue 2
EED: Embryonic Ectoderm Development
SUZ12: Suppressor of Zeste 12
RBBP4/7: Retinoblastoma-binding protein 4/7
RBBP5: Retinoblastoma binding protein 5
ASH2L: Absent, small, or homeotic 2-like
PRC2: Polycomb repressive complex 2
COMPASS: Complex Proteins Associated with Set1
KMTs: Lysine methyltransferases
ChIP-seq: Chromatin immunoprecipitation sequencing
CGI: CpG island
HMTs: Histone methyltransferases
HDMTs: Histone demethylases
GO: Gene Ontology
SYCP3: Synaptonemal complex protein 3
hBJ: Human BJ foreskin fibroblast
MEF: Mouse embryonic fibroblast
RA: Retinoic acid
*PARD3*: Partitioning defective 3 homolog
NGF: Nerve growth factor
hfNSCs: Human fetal neural stem cells
