# Supplementary figures and images for: H3K27me3-H3K4me1 transition at bivalent promoters instructs lineage specification in development
Source: Cell Biosci. 2023 Mar 29;13:66. doi: 10.1186/s13578-023-01017-3 (PMC10061859; doi:10.1186/s13578-023-01017-3)

A

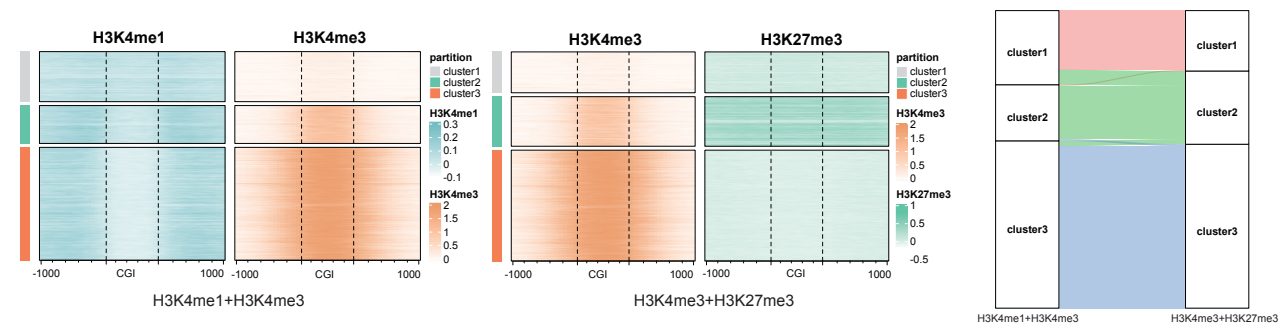

B

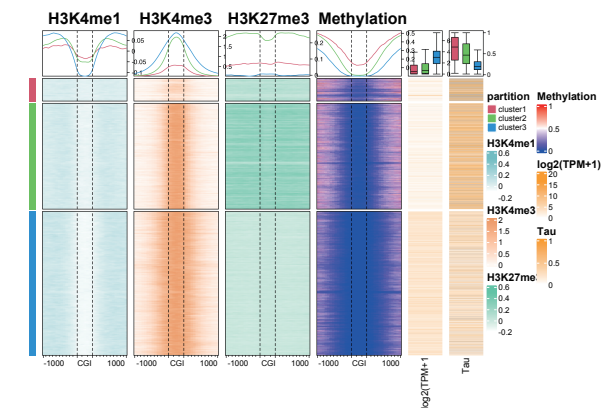

C

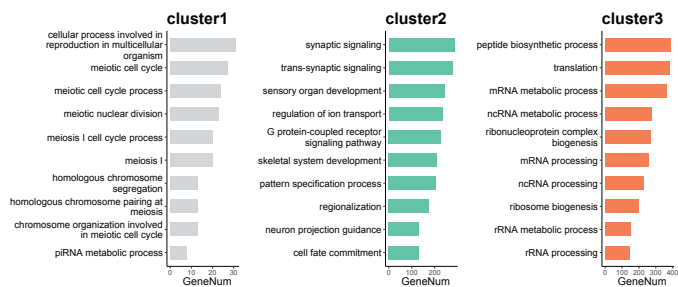

D

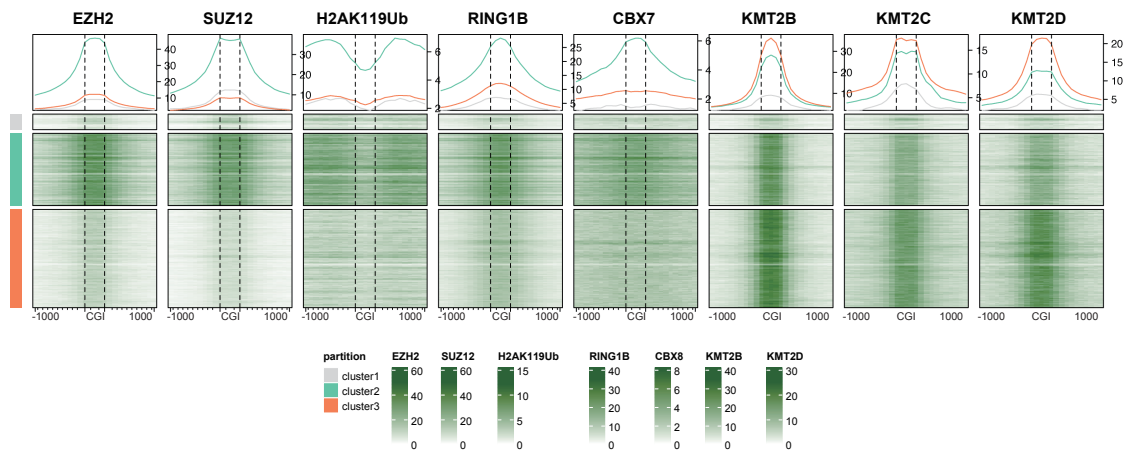

Supplement: Supplementary file 1 — Additional file 1: Figure S1. H3K4me1 in combination with H3K4me3 is able to partition promoters and predict bivalent promoters. A Heatmaps showing histone modifications patterns of promoter CGIs and their shores based on the distribution patterns of traditional bivalent marks (H3K4me3 and H3K27me3) (left) or non-traditional bivalent marks (H3K4me1 and H3K4me3) (right), respectively in hESCs; Sankey Diagram showing considerable overlaps between the clusters defined based on the two different bivalent marks combinations described above. B Heatmaps and average line plots showing histone modifications patterns and average methylation patterns of promoter CGIs and their shores in mESCs. Each line represents a single CpG island. Right panel: Heatmaps and boxplots showing gene expression and tissue-specific score (Tau) of three different clusters in mESC. C Histograms showing GO-term enrichment for genes involved in three clusters as in (B) in mESCs. D Heatmaps and average line plots showing H3K4 and H3K27 methylation related HMTs (EZH2, SZU12 for H3K27; KMT2B, KMT2C, KMT2D for H3K4; RYBP, CBX8 for H2AK119ub) patterns of promoter CGIs and their shores of three clusters as in (B) in mESCs, respectively. Each line represents a single CpG island. [file 13578_2023_1017_MOESM1_ESM.pdf]

**A**

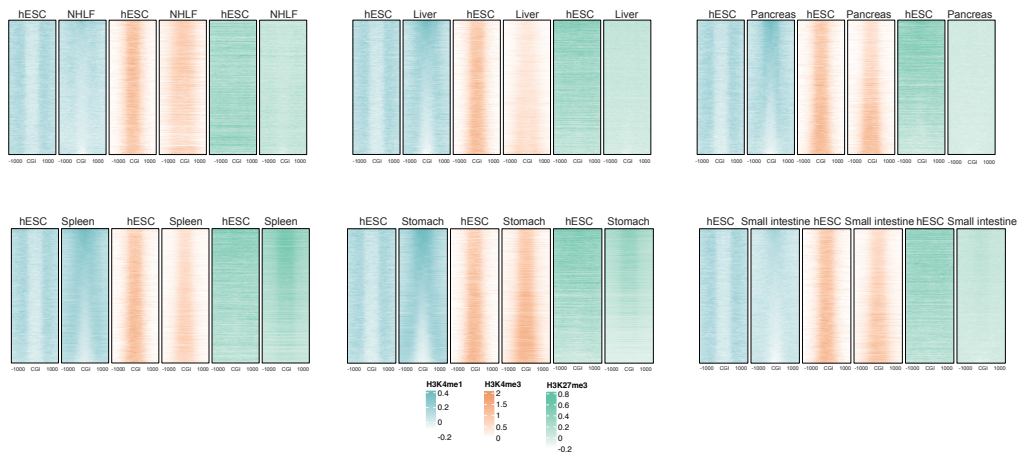

**B**

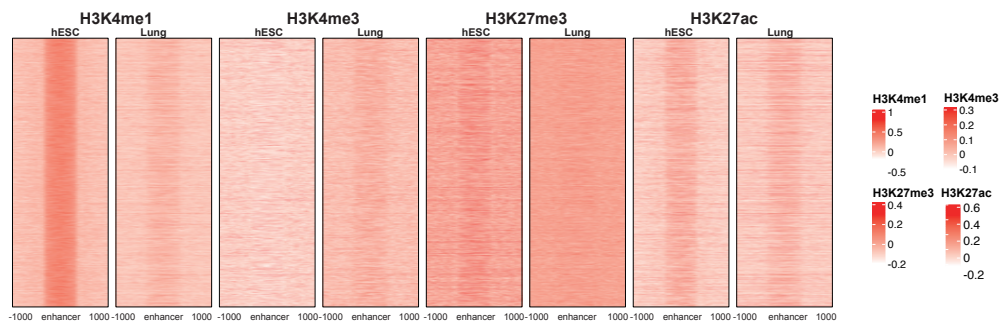

**C**

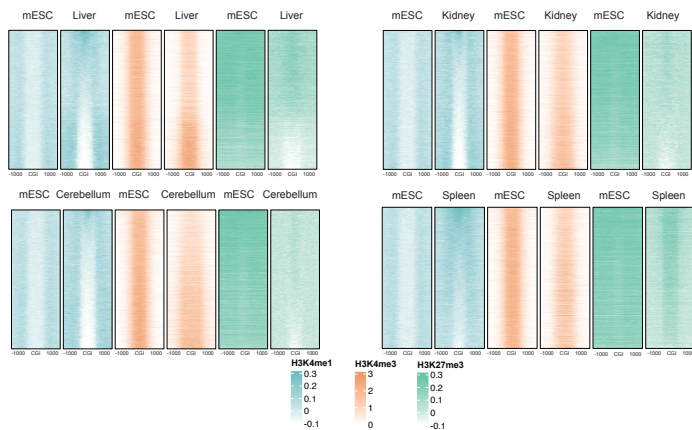

**D**

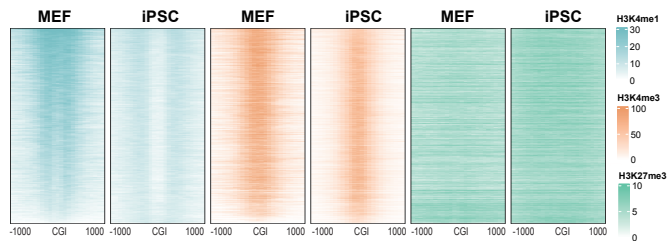

Supplement: Supplementary file 2 — Additional file 2: Figure S2. Bivalent promoter CGIs undergo H3K27me3-H3K4me1 transition during development. A Heatmaps showing H3K4me1, H3K4me3 and H3K27me3 patterns of promoter CGIs and their shores in bivalent cluster in hESC, normal human lung fibroblasts (NHLF) and other human tissues (liver, spleen, stomach, small intestine and pancreas). Each line represents a single CpG island. B Heatmaps showing H3K4me1, H3K4me3, H3K27me3 and H3K27ac patterns at enhancers in hESC and lung. Each line represents a single enhancer. C Heatmaps showing H3K4me1, H3K4me3 and H3K27me3 patterns of promoter CGIs and their shores in bivalent cluster in mESC and mouse tissues (liver, kidney, spleen and cerebellum). Each line represents a single CpG island. D Heatmaps showing H3K4me1, H3K4me3 and H3K27me3 patterns of promoter CGIs and their shores in bivalent cluster between mouse fibroblasts (MEF) and their reprogrammed iPSC cells. Each line represents a single CpG island. [file 13578_2023_1017_MOESM2_ESM.pdf]

**A**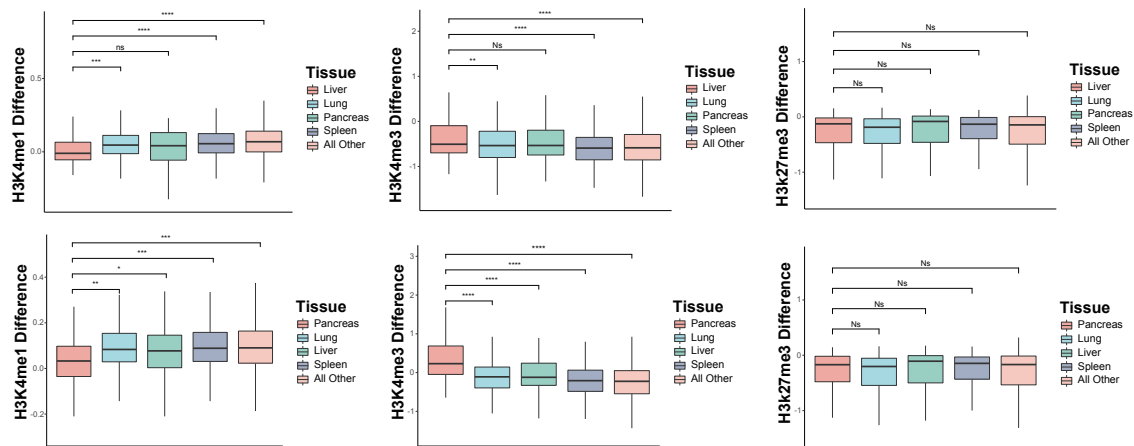**B**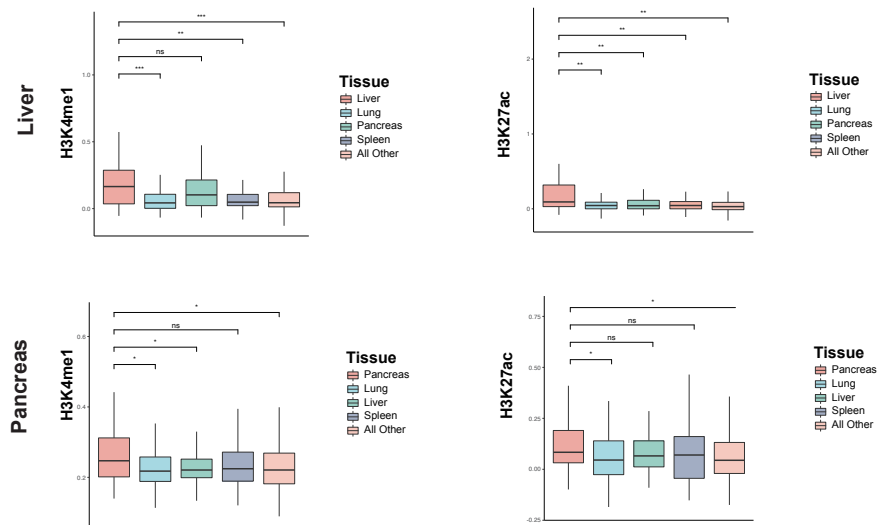**C**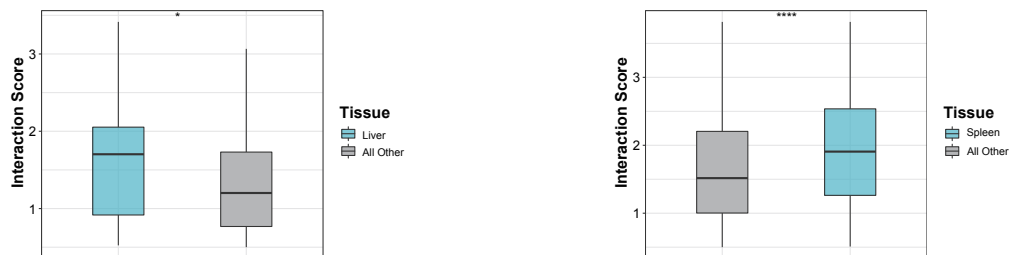

Supplement: Supplementary file 3 — Additional file 3: Figure S3. H3K27me3-H3K4me1 transition influences tissue-specific gene expression. A Top panel: boxplots showing the differences of H3K4me1, H3K4me3 and H3K27me3 on promoters of tissue specific genes between hESC and liver. All Other: all the other tissues without liver. Bottom panel: boxplots showing the differences of H3K4me1, H3K4me3 and H3K27me3 on promoters of tissue specific genes between hESC and pancreas. All Other: all the other tissues without pancreas. Lung: tissue-specific bivalent genes of lung; Liver: tissue-specific bivalent genes of liver; Pancreas: tissue-specific bivalent genes of pancreas; Spleen: tissue-specific bivalent genes of spleen. Significance was examined with t-test. *p value < 0.05, **p value < 0.01, ****p value < 0.0001﻿, ns: not significantly. B Top panel: boxplots showing levels of H3K4me1 and H3K27ac of tissue-specific bivalent genes at enhancers in liver. All Other: all the other tissues without liver. Bottom: boxplots showing levels of H3K4me1 and H3K27ac of tissue-specific bivalent genes at enhancers in pancreas. All Other: all the other tissues without pancreas. Lung: tissue-specific bivalent genes of lung; Liver: tissue-specific bivalent genes of liver; Pancreas: tissue-specific bivalent genes of pancreas; Spleen: tissue-specific bivalent genes of spleen. Significance was examined with t-test. *p value < 0.05, ****p value < 0.0001﻿, ns: not significantly. C Boxplot showing interaction score between promoter and enhancer of liver or spleen tissue-specific and all other (liver or spleen not included) tissue-specific bivalent genes in liver or spleen (Significance was examined with Wilcoxon rank-sum test, ****p value < 0.0001﻿). [file 13578_2023_1017_MOESM3_ESM.pdf]

**A**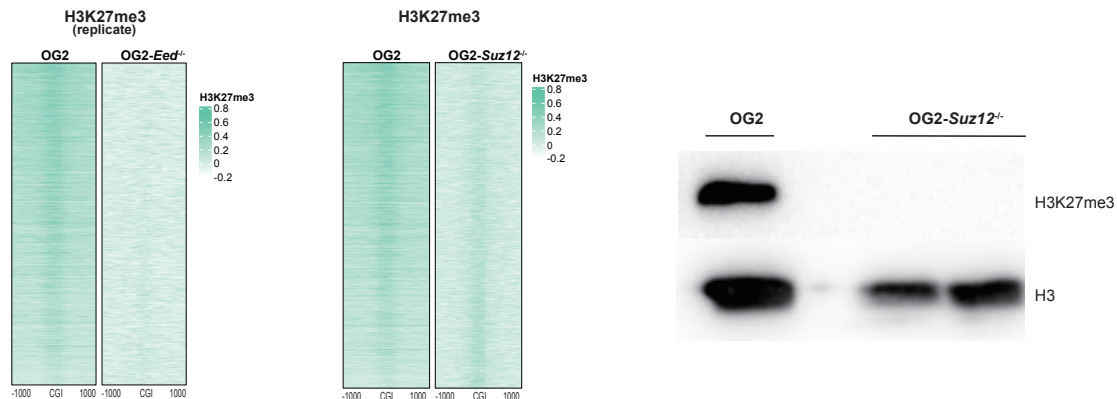**B**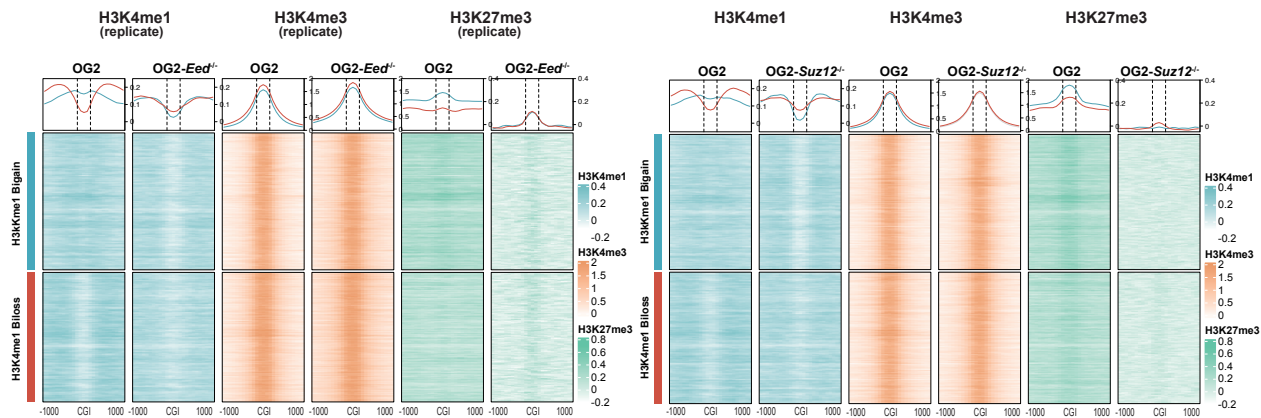**C**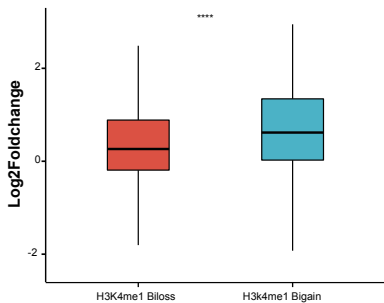**D**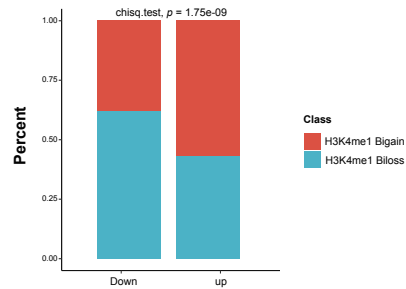

Supplement: Supplementary file 4 — Additional file 4: Figure S4. An artificial H3K27me3-H3K4me1 transition regulates the ESCs differentiation. A Western blotting analysis of H3K27me3 modification in WT and Suz12−/− OG2 mESCs. H3 as the loading control; heatmap showing H3K27me3 patterns of promoter CGIs and their shores in WT, Eed−/− (replicate) and SUZ12−/− OG2 mESCs. Each line represents a single CpG island. OG2: WT OG2 mESCs; OG2 Eed−/−: Eed−/− mESCs; OG2 Suz12−/−: Suz12−/− mESCs. B Heatmaps and average line plots showing H3K4me1, H3K4me3 and H3K27me3 patterns at promoter CGIs and their shores of two different groups (H3H4me1 biloss group and bigain group) in WT, Eed−/− (replicate) and SUZ12−/− OG2 mESCs. Each line represents a single CpG island. H3K4me1 Biloss: H3K4me1 bimodal-loss group; H3K4me1 Bigain: H3K4me1 bimodal-gain group. OG2: WT OG2 mESCs; OG2 Eed−/−: Eed−/− mESCs; OG2 Suz12−/−: Suz12−/− mESCs. C Boxplot showing expression alteration (log2Foldchange) of bivalent genes in H3K4me1 biloss group and H3K4me1 bigain group in Suz12−/− OG2 mESCs. Significance was examined with Wilcoxon rank-sum test, ****p value < 0.0001. H3K4me1 Biloss: H3K4me1 bimodal-loss group; H3K4me1 Bigain: H3K4me1 bimodal-gain group. D Histogram showing the percentage of H3K4me1 biloss group genes and H3K4me1 bigain group genes in the up-regulated (Up) or down-regulated (Down) genes in Suz12−/− OG2 mESCs compared with WT OG2 mESCs, respectively. Significance level was determined using ﻿χ2 tests, p value ﻿ = 1.159e-07. H3K4me1 Biloss: H3K4me1 bimodal-loss group; H3K4me1 Bigain: H3K4me1 bimodal-gain group. [file 13578_2023_1017_MOESM4_ESM.pdf]

**A**

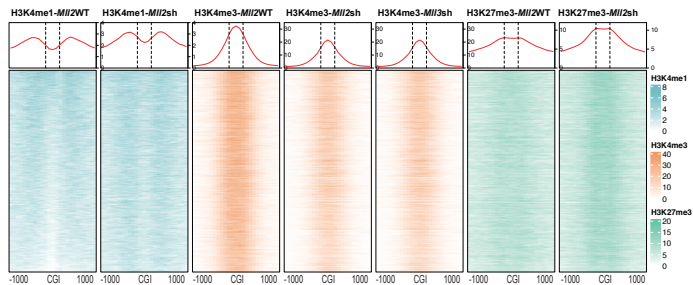

**B**

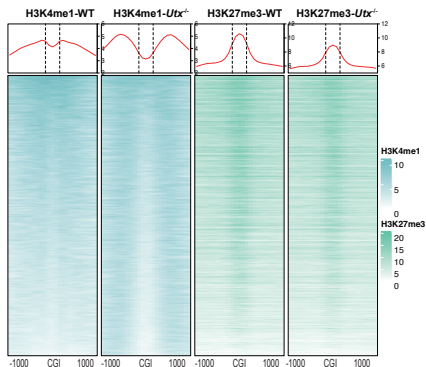

**C**

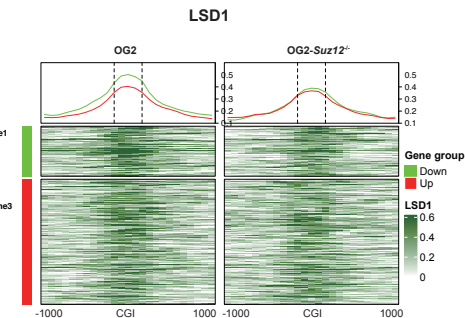

**D**

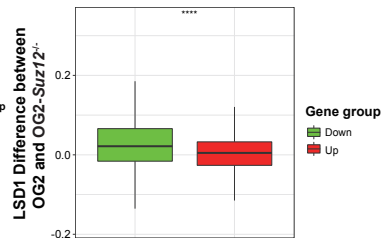

Supplement: Supplementary file 5 — Additional file 5: Figure S5. LSD1 interacts with core members of PRC2 and plays a significant role in the artificial H3K27me3-H3K4me1 transition in mESCs. A Heatmaps and ﻿average line plots showing histone modifications patterns of promoter CGIs and their shores in WT, Mll2-shRNA and Mll3-shRNA mESCs. Each line represents a single CpG island. B Heatmaps and ﻿average line plots showing histone modifications patterns of promoter CGIs and their shores in WT and Utx−/− mESCs. Each line represents a single CpG island. C Heatmaps and average line plots showing LSD1 patterns at promoter CGIs and their shores of up-regulated (Up) and down-regulated (Down) genes of bimodal-loss group in WT and Suz12−/− OG2 mESCs. Each line represents a single CpG island. OG2: WT OG2 mESCs; OG2 Suz12−/−: Suz12−/− mESCs. D Boxplot showing the differences of normalized LSD1 coverage at bivalent promoter CGIs between WT (OG2) and Suz12−/− (OG2 Suz12−/−) mESCs of up-regulated (Up) and down-regulated (Down) genes in bimodal-loss group. Significance was examined with t-test, ****p value < 0.0001. [file 13578_2023_1017_MOESM5_ESM.pdf]

**A**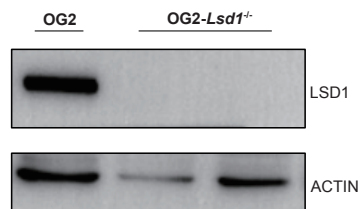**B**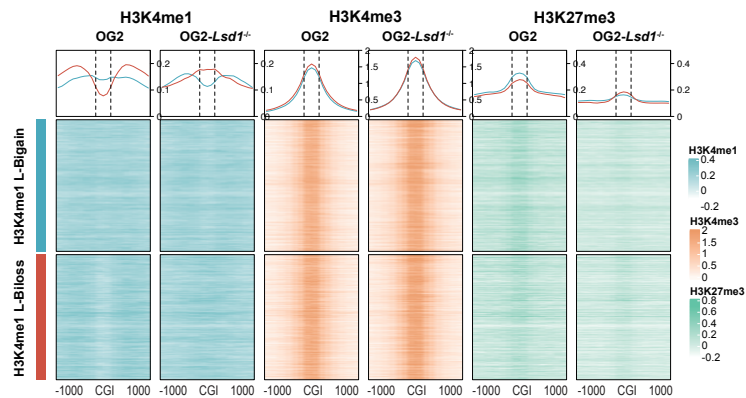**C**

*Lsd1* KO Trans CGIs

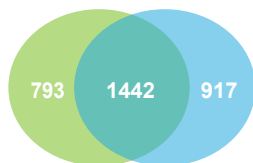

*Eed* KO Trans CGIs

**D**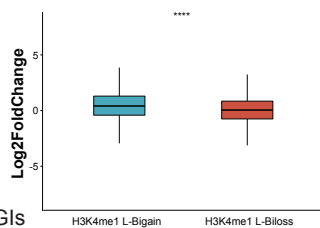**E**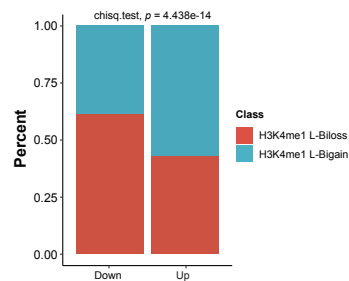**F**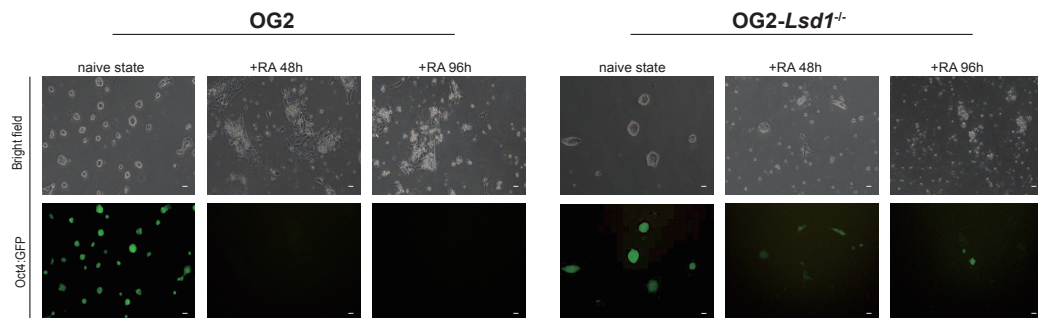

Supplement: Supplementary file 6 — Additional file 6: Figure S6. Lsd1 knockout induces similar effects as Eed or Suz12 knockout. A Western blot analysis of LSD1 protein in each indicated cell lines (OG2, OG2-Lsd1−/−). ACTIN as the loading control. B Heatmaps and average line plots showing H3K4me1, H3K4me3 and H3K27me3 patterns at promoter CGIs and their shores of two different groups in WT and Lsd1−/− OG2 mESCs. Each line represents a single CpG island. H3K4me1 L-Biloss: H3K4me1 L-bimodal-loss group; H3K4me1 L-Bigain: H3K4me1 L-bimodal-gain group. OG2: WT mESCs; OG2 Lsd1−/−: Lsd1−/− mESCs. C Veen diagram showing considerable overlaps of the promoter CGIs undergoing the artificial transition betweent Lsd1−/− and Eed−/− mESCs. ﻿Lsd1 KO Trans CGIs: the promoter CGIs undergoing an artificial transition in Lsd1−/− mESCs; Eed KO Trans CGIs: the promoter CGIs undergoing an artificial transition in Eed−/− mESCs. D Boxplot showing expression alteration (log2Foldchange) of bivalent genes in H3K4me1L- biloss group and H3K4me1 L-bigain group in Lsd1−/− OG2 mESCs. Significance was examined with Wilcoxon rank-sum test, ****p value < 0.0001. H3K4me1 L-Biloss: H3K4me1 L-bimodal-loss group; H3K4me1 L-Bigain: H3K4me1 L-bimodal-gain group. E Histogram showing the percentage of H3K4me1 L-bimodal-loss group genes and H3K4me1 L-bimodal-gain group genes in the up-regulated (Up) or down-regulated (Down) genes in Lsd1−/− mESCs compared with WT mESCs, respectively. Significance level was determined using χ2 tests, p value ﻿ = 4.438e-14. H3K4me1 L-Biloss: H3K4me1 L-bimodal-loss group; H3K4me1 L-Bigain: H3K4me1 L-bimodal-gain group. F Images showing the morphology of mESC cells after RA treatment. WT mESCs is OG2 mESCs with GFP expression controlled by Oct4 promoter (Oct4: GFP). Scale bar, 100 μm. OG2: WT mESCs; OG2 Lsd1−/−: Lsd1−/− mESCs. [file 13578_2023_1017_MOESM6_ESM.pdf]

**A**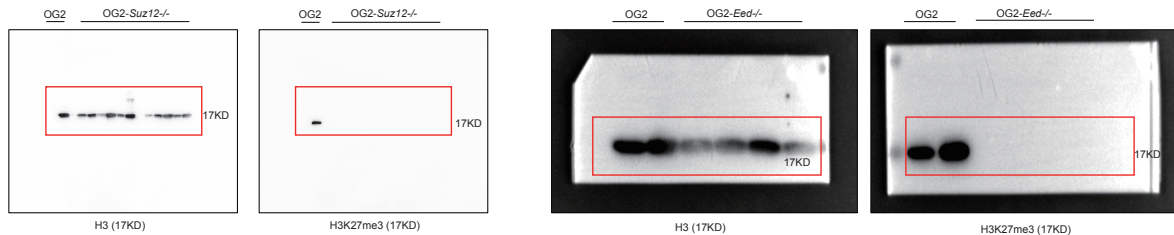**B**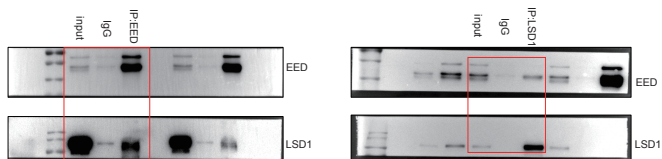**C**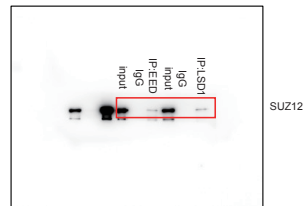**D**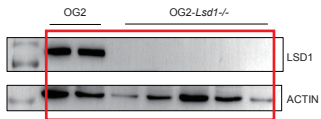

Supplement: Supplementary file 7 — Additional file 7: Figure S7. Uncropped scans of western blots. A Uncropped scans of western blots for Fig. 4A and Fig. S4A. Histone modification H3K27me3 level was analyzed by western-blot using the specific antibody on the whole cell lysates from indicated cell lines (OG2, OG2-Eed−/− and OG2-Suz12−/−). H3 as the loading control. Red box indicates the location of goal protein. KD Kilodaltons. B Uncropped scans of western blots for Fig. 5C. mESC cell nuclear extract was immunoprecipitated by an anti-LSD1 antibody, and subjected to western blotting analysis with anti-EED (left); mESC cell nuclear extract was immunoprecipitated by an anti-EED antibody, and subjected to western blotting analysis with anti-LSD1 (right). Red box indicates the location of goal protein. C Uncropped scans of western blots for Fig. 5C. mESC cell nuclear extract was immunoprecipitated by an anti-LSD1 or anti-EED antibody, and subjected to western blotting analysis with anti-SUZ12. Red box indicates the location of goal protein. D Uncropped scans of western blots for Fig. S6A. LSD1 expression level was analyzed in each indicated cell lines (OG2, OG2-Lsd1−/−). ACTIN as the loading control. Red box indicates the location of goal protein. [file 13578_2023_1017_MOESM7_ESM.pdf]
